# GC content of plant genes is linked to past gene duplications

**DOI:** 10.1371/journal.pone.0261748

**Published:** 2022-01-13

**Authors:** John E. Bowers, Haibao Tang, John M. Burke, Andrew H. Paterson

**Affiliations:** 1 Department of Plant Biology, University of Georgia, Athens, Georgia, United States of America; 2 Plant Genome Mapping Laboratory, University of Georgia, Athens, Georgia, United States of America; 3 Center for Genomics and Biotechnology, Key Laboratory of Ministry of Education for Genetics, Breeding and Multiple Utilization of Crops, Fujian Provincial Key Laboratory of Haixia Applied Plant Systems Biology, Fujian Agriculture and Forestry University, Fuzhou, Fujian, China; Huazhong Agriculture University, CHINA

## Abstract

The frequency of G and C nucleotides in genomes varies from species to species, and sometimes even between different genes in the same genome. The monocot grasses have a bimodal distribution of genic GC content absent in dicots. We categorized plant genes from 5 dicots and 4 monocot grasses by synteny to related species and determined that syntenic genes have significantly higher GC content than non-syntenic genes at their 5`-end in the third position within codons for all 9 species. Lower GC content is correlated with gene duplication, as lack of synteny to distantly related genomes is associated with past interspersed gene duplications. Two mutation types can account for biased GC content, mutation of methylated C to T and gene conversion from A to G. Gene conversion involves non-reciprocal exchanges between homologous alleles and is not detectable when the alleles are identical or heterozygous for presence-absence variation, both likely situations for genes duplicated to new loci. Gene duplication can cause production of siRNA which can induce targeted methylation, elevating mC→T mutations. Recently duplicated plant genes are more frequently methylated and less likely to undergo gene conversion, each of these factors synergistically creating a mutational environment favoring AT nucleotides. The syntenic genes with high GC content in the grasses compose a subset that have undergone few duplications, or for which duplicate copies were purged by selection. We propose a “biased gene duplication / biased mutation” (BDBM) model that may explain the origin and trajectory of the observed link between duplication and genic GC bias. The BDBM model is supported by empirical data based on joint analyses of 9 angiosperm species with their genes categorized by duplication status, GC content, methylation levels and functional classes.

## Introduction

DNA encodes the complexity of life, but one of the simplest statistics of a DNA sequence, the fraction of G+C nucleotides (GC content), is highly variable across the tree of life. The overall GC content of bacteria ranges from 13–75% [1] and eukaryotic nuclear genomes range from 20–60% [2]. In some eukaryotic species, GC content shows significant variation even within the same genome. In the human genome GC content of 20k base pair windows vary from 31–65% [3]. Significant local variation in GC content has been noted in honeybees, as well [4]. Among Angiosperm plants, GC content shows an irregular or bimodal distribution in the genes of monocot grasses while most dicots exhibit a more normal distribution [5, 6]. Localized differences in GC frequency in Eukaryotes have many proposed causes [7, 8] but a scientific consensus has not been reached.

Natural selection predicts the likely fate of many single base pair mutations, but when the fitness impact of mutations at a specific site approaches zero, their fate is more strongly influenced by the nearly neutral theory of evolution [9]. This means that, at sites without fitness impact, mutation will randomize the base composition of DNA sequences if given sufficient time. However, mutation rates are not balanced; the 12 possible substitution mutations (e.g., A→C, A→G … T→G) being separate chemical reactions each with their own rates that are independently influenced by various factors [2]. Given sufficient time, the base-pair composition of nucleotides at neutral sites will move toward equilibrium ratios determined by the relative rates of the different substitution mutation types. Since the sequenced strand is usually arbitrary, the nucleotide frequency simplifies to GC content, which is not impacted by C↔G and A↔T transversion mutations. While selection is certainly a factor contributing to the GC differences, variation in the rates of the 8 remaining mutation types substantially influence the nucleotide composition of genomes.

Increased frequencies of GC nucleotides have long been associated with hypomethylated sequences [10, 11], suggesting that methylation is inversely linked to GC content. Mutation of methylated cytosine (mC) to thymidine, often caused by UV light, is 10–50 times more likely than other substitutions in humans [12] and is a primary cause of skin cancers [13]. Some researchers have inferred that the driving factor for localized differences in GC content is cytosine methylation increasing C→T mutations [11], and depletion of GC in methylated genes. Direct support of the mutation hypothesis was found in 80 re-sequenced Arabidopsis genomes, showing a link between known methylation and C→T transitions [14]. Others have argued that the principal cause of GC content variation may be GC-biased gene conversion (gBGC) [15, 16]. gBGC occurs when heteroduplex DNA is created in a recombination event during meiosis, and incorrectly paired C = A pairs are replaced with C = G pairs. Over time, such a mechanism would increase the GC content. Different researchers suggest gene conversion vs. selection as primary causes of varying GC content [17]. The uncertainty may in part be due to different magnitudes of each mechanism in different species.

While the debate continues between gBGC and methyl-cytosine hypermutation as the primary cause of differing local GC content, the local magnitude of the two mechanisms is correlated. Cytosine methylation is associated with increases in nucleosome binding and reduced local recombination rates in Arabidopsis [18]. With reduced recombination rates, gene conversion is reduced, as the two processes are related [19]. Therefore, hypomethylated regions are more prone to recombination and GC-enriching gene conversion events, whereas hypermethylated regions are more prone to A/T-enriching substitutions. Both gBGC and hypermutation of methyl cytosine synergistically drive GC nucleotide frequency lower in methylated vs. hypomethylated regions. The relative impact of the two mechanisms is affected by the biology of the species involved. Species with long generation times would have reduced rates of meiosis dependent gene conversion, and variation in regional UV light levels as well as the relative exposure level of germline cells as determined by plant growth habit and form would impact the relative level of mutation of mC.

As DNA sequences are impacted by selection, any model solely relying on mutation is inadequate to accurately predict overall sequence biases in evolving genomes. A subset of the gene encoding sequences is presumed to be under substantially lower levels of selective constraint than the coding sequence average, specifically the third base pair of codons in protein encoding DNA. At the third codon position, most substitutions do not change the encoded amino acid, especially C↔T and G↔A transitions that include most SNP polymorphisms in plants [20, 21]. Codon bias, i.e. the relative frequencies of synonymous codons, appears largely due to GC3 content [22, 23]. Only 4 of 64 possible transitions in the third codon position change the encoded protein, along with slightly less than half (62/128) of the less frequent transversions (G↔C, G↔T, T↔A, and C↔A). Except for methionine, which is also the start codon of genes, any amino acid sequence can be encoded with 100% GC at the 3^rd^ bp of every codon, or with 0% GC.

While methylation and gene conversion frequency may be the direct factors that lead to localized differences in GC content between grass genes, they are not the ultimate cause. The reason(s) for persistent differences in local methylation and gene conversion rates must be explained. If high GC and low GC genes could be predicted by another seemingly independent characteristic of either group of genes, hints toward underlying cause(s) could be revealed. Here, we analyze GC content of syntenic vs. non-syntenic genes in nine Angiosperm species and consider its relationship to current methylation status. Syntenic genes are defined herein as those that occur in blocks that share closely corresponding order between two chromosomes. The species of interest include: rice (*Oryza sativa*; [24], sorghum (*Sorghum bicolor*; [25], maize (*Zea mays*; [26], purple false brome (*Brachypodium distachyon*; [27], mouse ear cress (*Arabidopsis thaliana*; [20], poplar (*Populus trichocarpa*; [28], peach (*Prunus persica*; [29], grape (*Vitis vinifera*; [30], and tomato (*Solanum lycopersicum*; [31].

We propose to examine if some types of gene duplications are correlated with GC content in the 9 species involved. Genes for which gene duplication is either favorable or fitness-neutral may persist for some time, allowing interactions between copies to alter mutational rates and spectra. Conversely, genes for which duplicate copies reduce fitness, would be rapidly purged of duplications with minimal time for such interactions. Ascertaining if a gene has undergone duplication in its evolutionary past is challenging—many gene duplications are eventually lost, as the unduplicated state was sufficient for survival in the ancestor. Reconstructing complete long-term gene duplication history of all genes is problematic, as the ancestors are long extinct and/or sister taxa (even if they exist) are only approximate representations of ancestral states. We can however identify genes that are in their ancestral location by using synteny, and therefore are not new copies resulting from non-tandem duplications.

The use of synteny allows targeted examination of a subset of gene duplication events which may provide hints to underlying mechanisms. Gene duplications resulting from polyploidy are unlikely to be a primary cause of differential GC enrichment as polyploidy is infrequent and as all genes are duplicated at least initially. Tandem gene duplicates may permit continued recombination events and gene conversion of new copies. Genes classified as either syntenic or non-syntenic, respectively, could in theory have been subjected to different levels of recombination related gene conversion or cytosine methylation.

When comparing genomes that have diverged for millions of years, the subset of genes that are not syntenic are more likely to be lost in one lineage or have undergone duplication by non-tandem mechanisms followed by loss of the original copy. Syntenic genes between distantly related genomes can be presumed to be advantageous; [32] otherwise they would have been lost or degenerated by mutation. Syntenic genes also tend to be single copy genes, as gene families that have expanded since the last common ancestor (except by tandem duplication) would have copies in non-syntenic positions. The non-syntenic genes are more likely to be subject to presence-absence variation within the species, as new genes in growing gene families are likely to be variable within individuals of a species. Genes that duplicate can appear to move around in the genome over time, as new redundant gene copies can permit loss of the ancestral copy while maintaining function. Consequently, status of whether a gene is syntenic across species might be used as a proxy for the duplicability of a gene. The binary classification of genes into syntenic and non-syntenic groups will not perfectly correspond to duplicated and non-duplicated genes, but it is a close practical approximation. We propose herein a model as an addition to, not a replacement for, the six models influencing duplicate gene retention already proposed [33].

## Results

### Identification of syntenic genes

**Fig 1:**
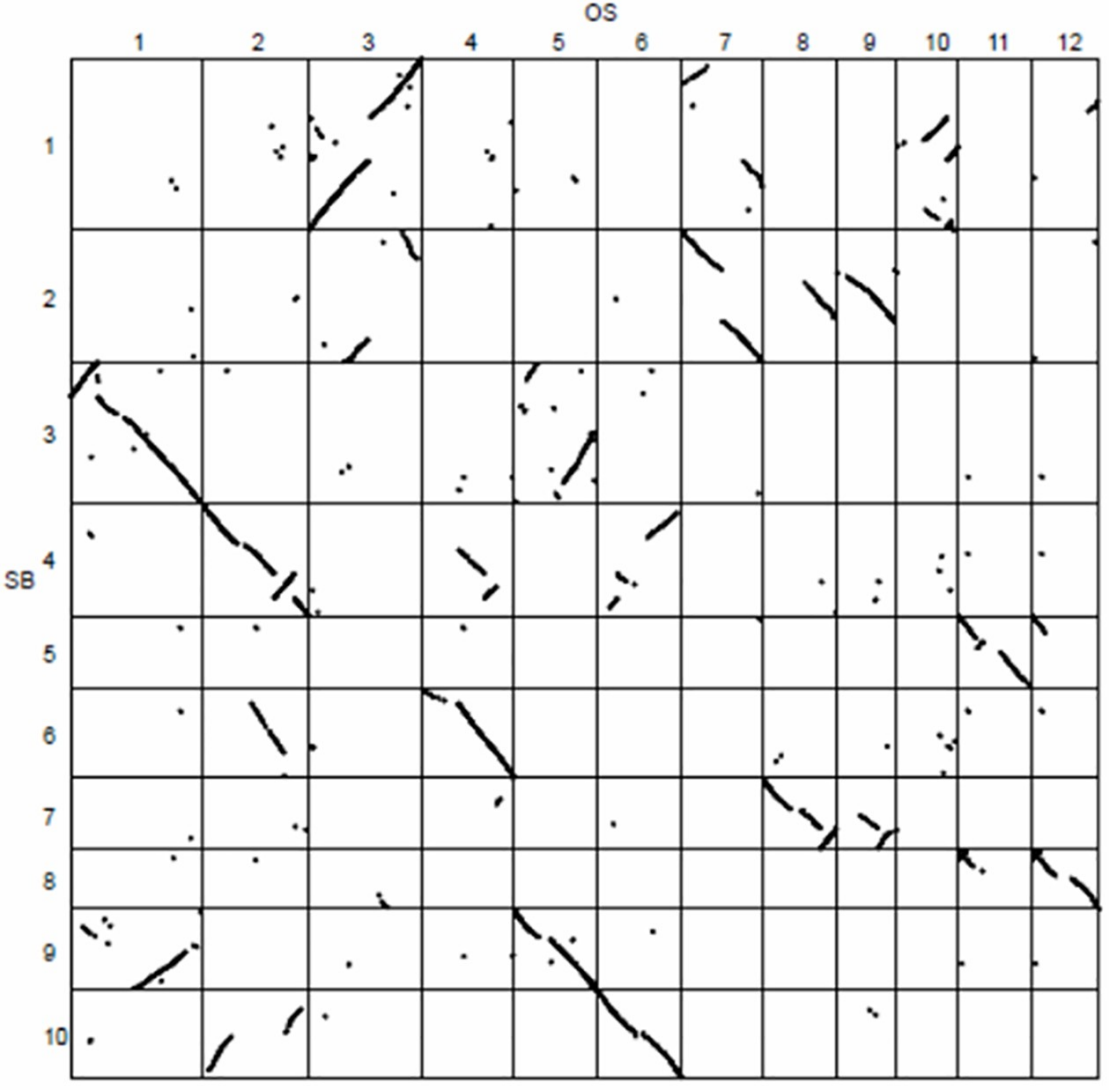
Sorghum to rice synteny. Dots indicate syntenic gene pairs conserved in gene order between the two species. Syntenic segments were identified as stretches of 7 or more genes conserved in order between the two genomes. The x-axis represents 52,424 rice genes and the y-axis represents 47,205 sorghum genes.

**Fig 2:**
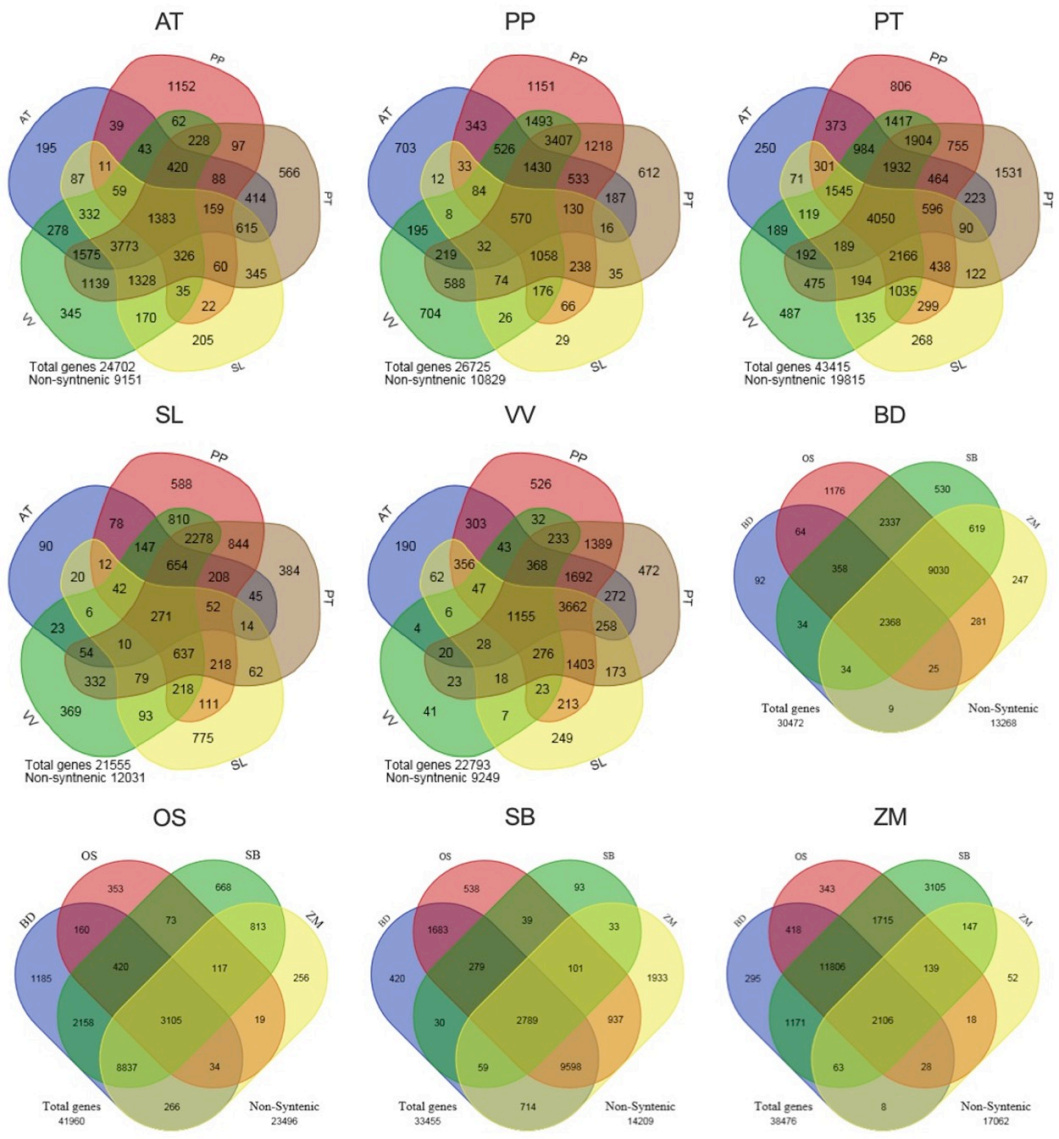
Number of genes from 9 species found to be in a syntenic location in other species. Number of genes from 9 species found to be in a syntenic location in other species. AT- *Arabidopsis thaliana*, PT*- Populus trichocarpa*, PP*- Prunus persica*, SL- *Solanum lycopersicum*, VV- *Vitis vinifera*, BD- *Brachypodiuum distachyon*, OS- *Oryza sativa*, SB*- Sorghum bicolor*, ZM- *Zea mays*.

To identify genes that have been repeatedly duplicated, we examined the annotated genes from the genomes of nine seed plants, including five eudicots and four monocots. Within each of these two groups, pairwise gene order synteny was assessed between all species pairs as well as by self-comparisons within genomes. Pairwise synteny was visualized using dot plots of gene orders between species followed by automated detection of significant clusters of shared gene orders (Fig 1, S5 File). Genes in syntenic segments in one or more comparisons were classified as remaining in their ancestral location, while all others were considered non-syntenic genes and inferred more likely to be new duplicated copies or novel, lineage-specific genes. The synteny status of the genes for each species is summarized in Fig 2, and the raw data, gene number and synteny is summarized is in S1 and S2 Files.

Among the grasses, 44–57% of genes had syntenic matches in one or more species, with the best matching genome pairs (sorghum-maize and rice-*Brachypodium*) being consistent with phylogenetic relationships. In eudicots, 41–59% of genes from each species had syntenic matches in one or more genome comparisons. Synteny in comparisons of a species to itself, showing duplicated genes retained in the ancestral position since the most recent polyploidy events, ranged from 6.6% to 35.3%. Synteny among gene orders arising from ancient polyploidy events was also revealed by comparison to other genomes that shared those events. In both monocots and eudicots, genes found to be syntenic in one comparison were more likely to be syntenic in other comparisons (Fig 2). Grape and peach, which share the same ploidy level relative to their last common ancestor, showed the highest fractions of genes identified as syntenic between species. Tomato and poplar have each experienced lineage-specific polyploidy events since their divergence and, accordingly, self-comparisons showed higher fractions of syntenic genes.

### Characterization of gene GC content

**Fig 3:**
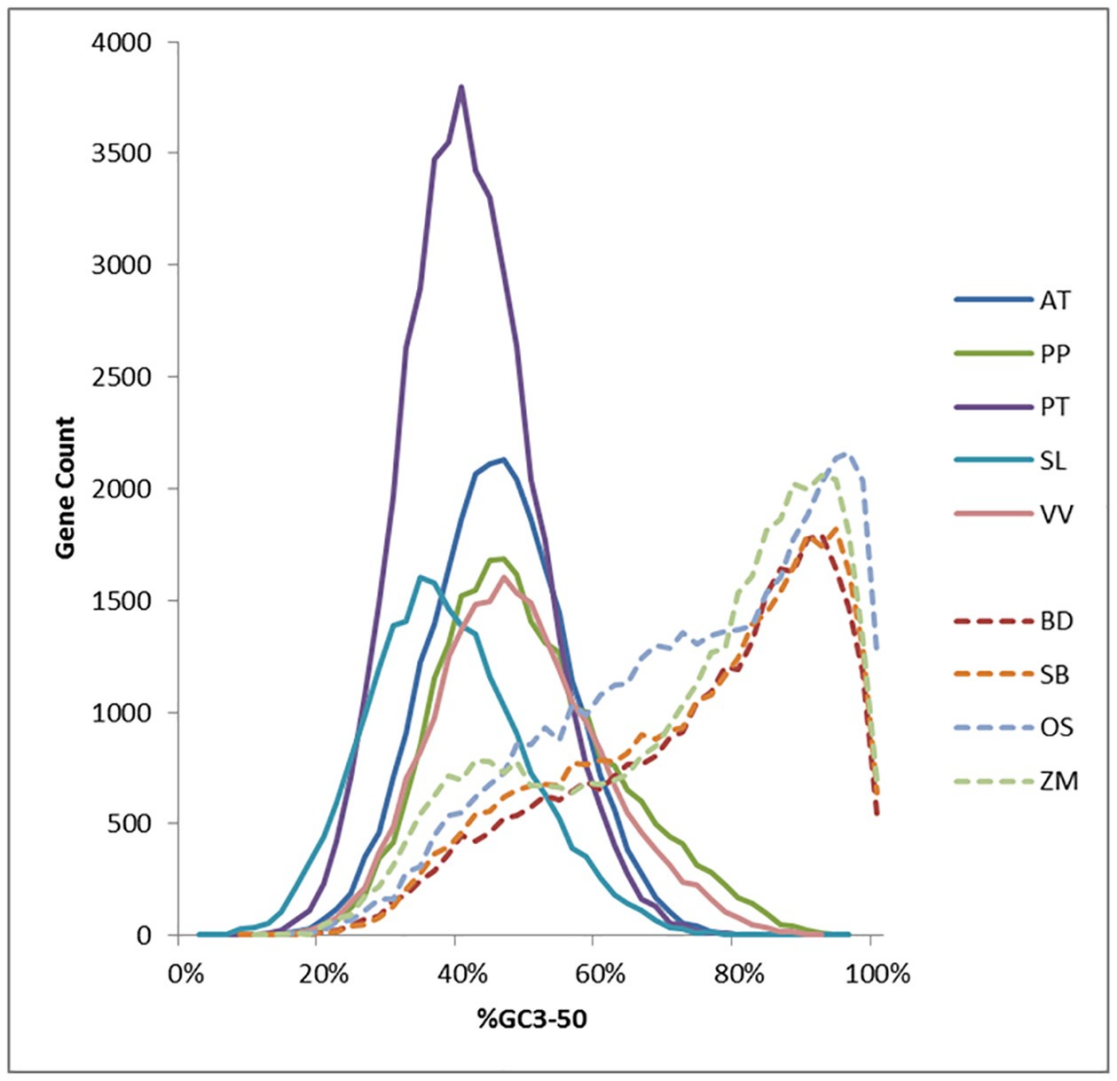
Plot of the GC content of all genes at the 3^rd^ position of each codon for the first 50 codons (GC3-50). The GC3-50 content of genes showed a very different distribution among the monocot grasses tested vs the eudicots tested. The eudicots appeared to have a relatively normal distribution of GC3-50 content, with *Arabidopsis*, peach and grape having median content of approximately 46% but poplar and tomato showing lower medians of 40% and 34% respectively. The grasses had irregular distributions of GC3-50 content, containing a class of genes with a modal GC3-50 content of 90–94%, and another class with a mode near 40% with many genes falling in between the two clusters. AT- *Arabidopsis thaliana*, PT*- Populus trichocarpa*, PP*- Prunus persica*, SL- *Solanum lycopersicum*, VV- *Vitis vinifera*, BD- *Brachypodiuum distachyon*, OS- *Oryza sativa*, SB*- Sorghum bicolor*, ZM- *Zea mays*.

To determine the nucleotide composition at sites relatively unbiased by selection, we calculated the average GC content of the third position of codons (GC3) within gene-encoding sequences. As average GC3 content in plant genes decreases with distance from the start codon (S5 File); [16], individual gene GC3 content is impacted by gene length. Therefore, whole gene GC3 content is strongly correlated with gene length. To create a length-independent measure, we computed the average GC3 content for only the first 50 codons (GC3-50) of each gene. Whole gene GC3 content is correlated with synteny in the grasses but appears less strongly correlated to synteny than GC3-50. We chose to use GC3-50 (which is the minimum predicted gene length for several of the species), to disambiguate GC3 content from variation in gene length. The distribution of GC3-50 in all annotated genes (Fig 3) for the five eudicot species is bell-shaped with averages ranging from 34% to 46%. For the four grass species, however, the distribution of GC3-50 is more complex, with one broad peak or shoulder near the dicot average (40–50%) and a peak near 94%.

### Synteny and GC content correlation

**Fig 4:**
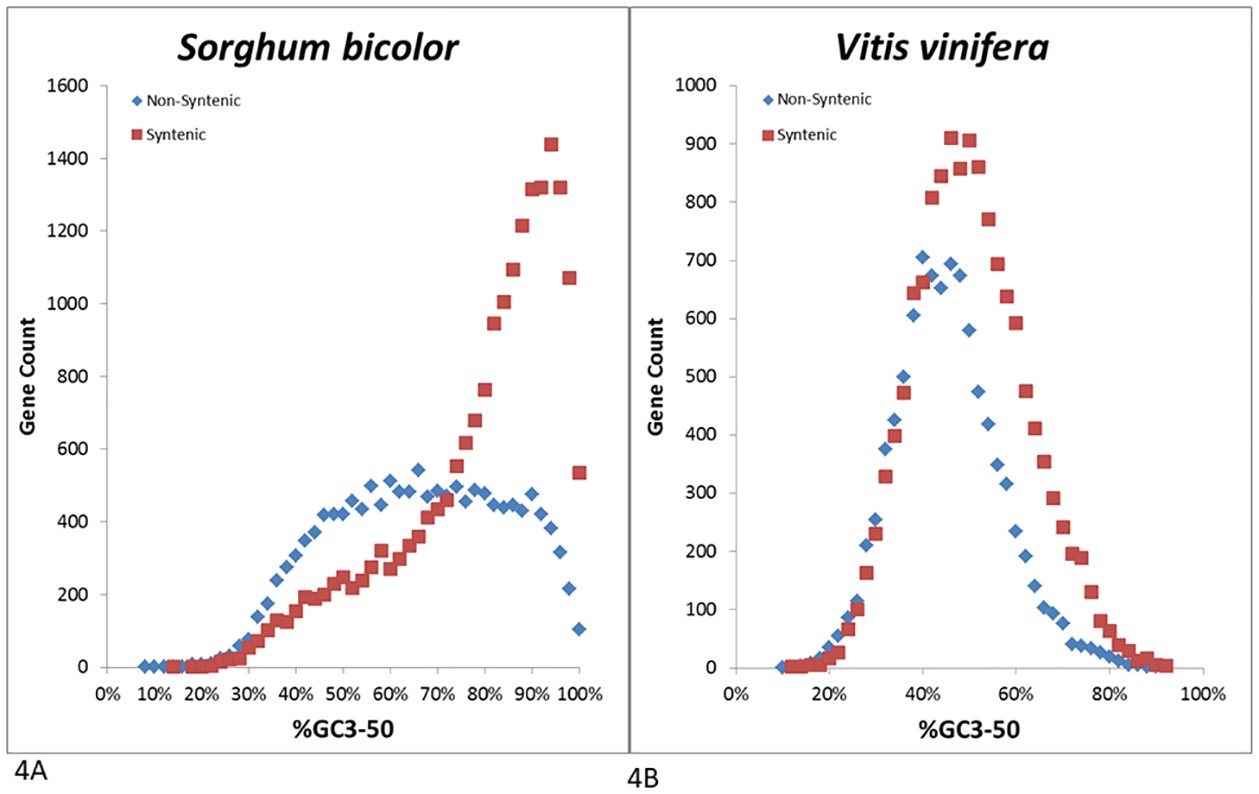
A. Synteny status of genes by percent GC content at the third position for the first 50 codons (%GC3-50) for 14,209 non-syntenic and 19,246 syntenic *Sorghum bicolor* genes.B. Synteny status of genes by percent GC content at the third position for the first 50 codons (%GC3-50) for 13,544 syntenic and 9,249 non-syntenic *Vitis vinifera* genes.

To investigate the relationship between GC content and synteny, we clustered the genes from each species according to GC3-50 and counted the number of syntenic and non-syntenic genes in each bin. Syntenic genes within the grasses had 10.8–13.0% higher average GC3-50 than non-syntenic genes (Fig 4A, S5 File). Syntenic genes for all four grass species were primarily distributed between 80–100% GC3-50, while the non-syntenic genes had relatively broad, possibly multi-modal, distributions with GC3-50 ranging from 40–90%. The difference between syntenic and non-syntenic genes was highly significant (*P* < 0.0001, Wilcoxon rank-sum test) for all species separately. The comparison for the five eudicot species was less clear, as the distribution of syntenic and non-syntenic genes largely overlapped (Fig 4B, S5 File). For all five eudicot species, the average GC3-50 for syntenic genes was higher than for non-syntenic genes by 0.8–4.9%. In contrast to the grasses, the GC3-50 of the five eudicots appeared to show relatively normal distributions for both syntenic and non-syntenic genes. While the GC3-50 difference between syntenic and non-syntenic genes was less pronounced than for the grass species, it was still highly significant (*P* < 0.0001, Wilcoxon rank-sum test) for all species.

**Fig 5:**
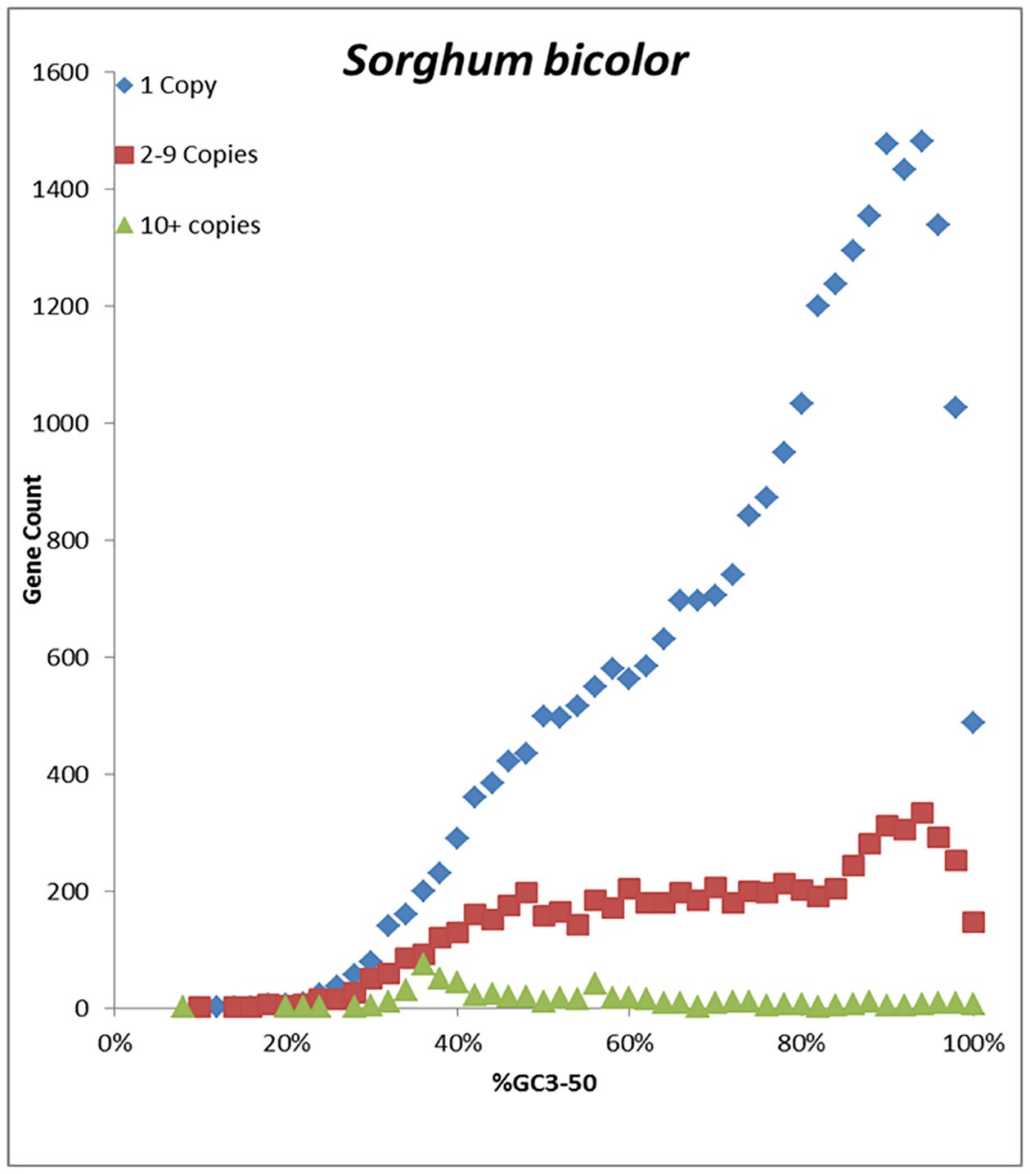
Gene count distribution by %GC3-50 and copy number of gene within the genome assembly by number of high similarity BLAST hits for 26,298 single copy, 6,954 moderately repetitive and 558 repetitive *Sorghum bicolor* genes.

The within-genome copy number of genes was determined by counting high similarity BLAST hits of the first 150 base pairs of genes to the genome sequence. The BLAST hit copy number was binned into three classes (i.e., single copy, 2–9 copies, and 10+ copies) which were found to be correlated with GC3-50 (Fig 5, S5 File). For both grasses and eudicots, single copy genes showed similar distributions to the syntenic fraction, and the moderately repetitive genes resembled the non-syntenic fraction seen in Fig 5 for the same species, while the most repetitive genes had the lowest mean GC3-50. The difference in GC3-50 of single and multicopy genes was significant (*P* < 0.0001, Wilcoxon rank sum test) for eight of the nine species, excluding poplar. Overall, the trend for all 9 species is that syntenic genes are less frequently duplicated and tend to have higher GC3-50 content.

### GO term enrichment of syntenic and non-syntenic classes

**Fig 6:**
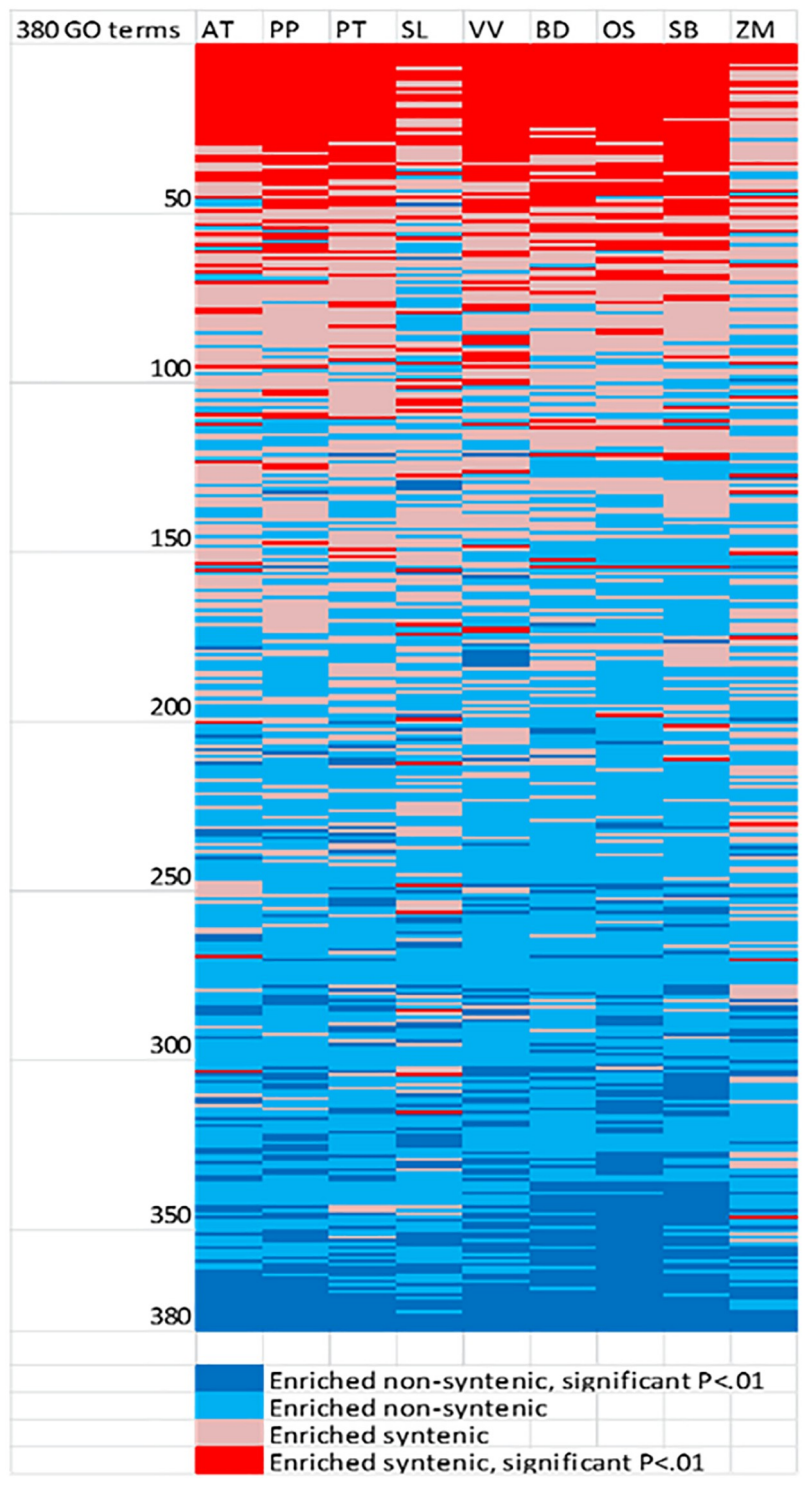
Evaluation of 380 GO terms with at least 20 genes for enrichment in each species for syntenic and non-syntenic genes across nine species. A total of 46 GO terms were significantly enriched for syntenic genes for five or more species, while 33 were significantly enriched for non-syntenic genes for five or more species.

Syntenic and non-syntenic gene classes were enriched for different gene ontology (GO) terms. The genomes of each of the nine species individually were significantly (*P* < 0.01) enriched for six GO terms in the syntenic class, and a different five GO terms in the non-syntenic class. A further 23 GO classifications were significantly enriched for 8 of 9 species (Fig 6, S4 File). Many other GO classifications were significantly enriched for several of the species tested for either the syntenic or non-syntenic categories, with 79 GO terms showing significant enrichment for 5 or more species.

Four of the 5 of the GO terms enriched for non-syntenic genes included transcription factors, or related functions (transcription, DNA-templated; regulation of transcription, DNA-templated; transcription factor activity, sequence-specific DNA binding; transcription regulatory region DNA binding). Other terms enriched for non-syntenic genes included many defense related categories, although some GO terms associated with defense response were enriched in the syntenic class as well. Three of the six terms enriched in the syntenic category were for binding proteins (ATP binding; heme binding; ADP binding). Other GO terms enriched for syntenic genes in multiple species included some basic functions such as cell walls or metabolic processes.

### Comparison to current methylation status

The methylation status of the first 150 bp of the genes at CG, CHG, and CHH sites were investigated for all nine species using whole genome bisulfite sequencing data. The average methylation at CG, CHG, and CHH sites was significantly higher for non-syntenic than syntenic genes for all nine species (Table 1). The average level of methylation at CG, CHG, and CHH sites also showed an upward trend with sequence copy number within the genome for all nine species tested (Table 2). This agrees with a previous study that found syntenic genes in maize were less likely to be methylated than non-syntenic genes [34].

**Table 1:** Average methylation level at CG, CHG and CHH sites for syntenic and non-syntenic genes for nine plant species.

Species	Syntenic?	Average methylation level		
		CG	CHG	CHH
*Arabidopsis*	Non-Syntenic	11.46%	3.84%	1.57%
*thaliana*	Syntenic	6.80%**	0.71%**	0.62%**
*Prunus*	Non-Syntenic	28.26%	12.22%	1.90%
*persica*	Syntenic	21.12%**	4.00%**	1.05%**
*Populus*	Non-Syntenic	20.31%	6.51%	1.38%
*tricocarpa*	Syntenic	8.02%**	1.09%**	0.45%**
*Solanum*	Non-Syntenic	17.05%	4.27%	2.25%
*lycopersicum*	Syntenic	12.08%**	2.24%*	1.44%*
*Vitis*	Non-Syntenic	32.80%	13.19%	0.74%
*vinifera*	Syntenic	26.19%**	1.69%**	0.34%**
*Brachypodium*	Non-Syntenic	31.30%	11.95%	0.84%
*distachyon*	Syntenic	20.04%**	1.50%**	0.47%**
*Oryza*	Non-Syntenic	41.34%	23.51%	3.61%
*sativa*	Syntenic	14.00%**	2.42%**	0.93%**
*Sorghum*	Non-Syntenic	21.55%	7.24%	1.10%
*bicolor*	Syntenic	13.20%**	0.88%**	0.52%**
*Zea*	Non-Syntenic	29.01%	19.93%	0.79%
*mays*	Syntenic	10.11%**	1.68%**	0.43%**

** Significantly different at *P* ≤ 0.01 by Wilcoxon ranked sum test,

* Significantly different at *P* ≤ 0.05 by Wilcoxon ranked sum test.

**Table 2:** Average methylation levels at CG, CHG and CHH sites by gene copy number as determined by number of within genome BLAST hits.

Species	Copy #	cg	chg	chh
*Arabidopsis*	1 copy	7.49%	1.36%	0.82%
*thaliana*	2–9 copies	14.78%	5.22%	1.99%
	10+ copies	75.22%	25.62%	9.78%
*Prunus*	1 copy	18.98%	2.55%	0.94%
*persica*	2–9 copies	27.61%	11.28%	1.84%
	10+ copies	77.04%	54.37%	5.18%
*Populus*	1 copy	12.60%	2.47%	0.69%
*tricocarpa*	2–9 copies	13.62%	3.57%	0.85%
	10+ copies	29.20%	26.56%	5.03%
*Solanum*	1 copy	13.29%	2.44%	1.59%
*lycopersicum*	2–9 copies	18.15%	6.24%	2.53%
	10+ copies	42.75%	36.83%	17.16%
*Vitis*	1 copy	26.48%	2.77%	0.38%
*vinifera*	2–9 copies	32.63%	12.82%	0.73%
	10+ copies	54.98%	36.16%	1.42%
*Brachypodium*	1 copy	20.89%	3.18%	0.57%
*distachyon*	2–9 copies	38.81%	15.87%	0.83%
	10+ copies	78.03%	47.34%	1.63%
*Oryza*	1 copy	22.66%	9.21%	2.03%
*sativa*	2–9 copies	37.28%	19.36%	3.02%
	10+ copies	85.77%	62.37%	5.26%
*Sorghum*	1 copy	14.10%	2.12%	0.70%
*bicolor*	2–9 copies	25.52%	7.99%	0.94%
	10+ copies	56.23%	30.06%	1.74%
*Zea*	1 copy	10.38%	3.12%	0.49%
*mays*	2–9 copies	22.30%	11.62%	0.57%
	10+ copies	61.37%	49.02%	1.36%

**Fig 7:**
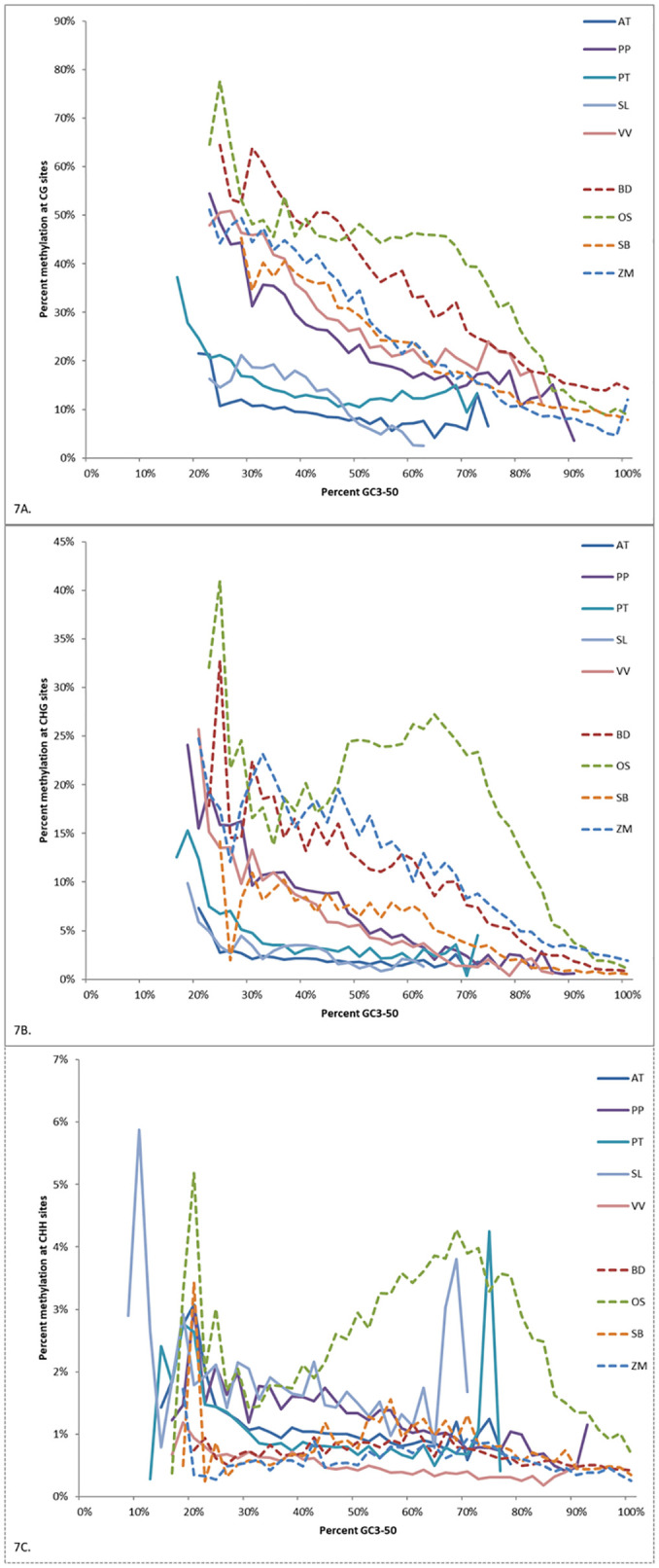
A. Average methylation level at CG sites for genes with different levels of GC3-50 for nine species. Only bins with a minimum of 200 CHG sites shown. B. Average methylation level at CHG sites for genes with different levels of GC3-50 for nine species. Only bins with a minimum of 200 CHG sites shown. C. Average methylation level at CHH sites for genes with different levels of GC3-50 for nine species. Only bins with a minimum of 200 CHG sites shown.

Methylation levels at CG, CHG, and CHH sites were binned according to GC3-50 and plotted (Fig 7). The average methylation levels showed significant negative correlations with GC3-50 levels for all species tested (Table 3). For CHG and CHH, higher methylation levels occurred mainly among a small subset of genes, usually with low GC3-50 values, except rice. A few individual genes in poplar and tomato were highly CHH methylated despite having higher GC3-50 percentages, producing narrow peaks contrary to the overall trends. There was significant negative correlation between methylation levels of all types and GC3-50 content for all nine species tested (Table 3). This gradient in methylation may be sufficient over time to induce at least part of the correlated GC bias.

**Table 3:** Correlation between methylation levels for three different methylation types and the GC3-50 content for genes from nine species.

Species	Correlation between GC3-50 content and		
	CG methylation	CHG methylation	CHH methylation
*Arabidopsis*	‒0.066**	‒0.040**	‒0.033**
*Prunus*	‒0.179**	‒0.161**	‒0.059**
*Populus*	‒0.062**	‒0.058**	‒0.045**
*Solanum*	‒0.152**	‒0.076**	‒0.034*
*Vitis*	‒0.192**	‒0.151**	‒0.078**
*Brachypodium*	‒0.302**	‒0.272**	‒0.062**
*Oryza*	‒0.351**	‒0.283**	‒0.091**
*Sorghum*	‒0.262**	‒0.217**	‒0.054**
*Zea*	‒0.351**	‒0.240**	‒0.034**

** Significant at *P* ≤ 0.0001,

* Significant at *P* ≤ 0.05.

## Discussion

### Biased gene duplication and biased mutation

The correlation between GC3-50 and synteny status of plant genes, especially in the grasses, suggests pathway(s) linking these two characteristics. When individual genes are involved in non-tandem duplication, the new copy is non-syntenic, and both copies are redundant, so the original (syntenic) copy can be lost by mutation with little or no fitness impact. Repeated gene duplications and loss in either lineage will make it increasingly likely that members of a gene family will not be syntenic. Genes that remain syntenic with distantly related genomes represent a subset of the gene-space that is less duplication prone, or instances in which the duplicate copies were selected against and quickly lost possibly due to gene dosage balance [35]. It follows that plant genes that have undergone more non-tandem gene duplications have experienced a different mutational environment than genes that have remained single copy and syntenic.

The bimodal distribution of GC content in the grasses is unlikely to be due to selection, as most 3^rd^ base in a codon changes do not change the encoded protein. While gene expression levels [8] and/or tissue specificity [36] show a significant correlation with GC content of grass genes, the effect appears to be limited. While selection is likely to play a limited role [37, 38], GC content is the principal factor determining codon bias in a wide range of species [39].

In plant genomes, C↔T transitions typically account for most observable SNPs. For example, 70% of SNPs in rice [21] and 52.1% in *Arabidopsis* [20] are C/T polymorphisms. Since 15% and 25.2% of other SNPs involve A/T or C/G polymorphisms in rice and *Arabidopsis*, respectively, the C↔T mutation rates are the prime factor determining C/G frequency in the absence of selection. C↔T polymorphisms are enhanced by both gene conversion and elevated mutation associated with methylated cytosine, so either mechanism or both synergistically could cause the observed local variation in GC content.

### The role of methylation

A portion of methylation in plant genomes can be related to the silencing of duplicated gene copies [40]. When genes are duplicated, one copy will sometimes produce siRNAs that can methylate and silence (or at least reduce) the expression of one or both copies via RNA-directed DNA methylation (RdDM) [41]. The reduced expression level of the methylated gene may still be adequate for the plant’s needs, or the gene may be activated by loss of methylation in certain tissues [42]. This provides a mechanism to fine-tune gene expression levels in the interest of maximizing fitness. Gene silencing (or attenuation) would only be advantageous for some genes, while silencing of other genes may reduce protein levels below required levels. For example, methylation can be reduced due to stress [43, 44] so siRNA silencing could be a mechanism to reversibly inactivate conditionally advantageous genes. In this case, the retention of selectively advantageous siRNA-producing copies would experience purifying selection, but if RdDM reduces fitness, the presence of siRNA-producing copies would be selected against.

RdDM works when siRNAs find a complementary match in the target genes but is lost as mutation causes divergence between the siRNA and its target. The immediate selection against diverging mutations might, however, be limited, as methylation can be maintained in plants at CmG and CmNG sites for multiple generations [45]. The maintenance of methylation also means that an siRNA-producing gene copy could prove beneficial to a larger population—i.e., not just the individuals with the siRNA-producing copy, but also potentially their descendants lacking the siRNA producing sequence. But methylation at CG and CNG sites is replicated with lower fidelity than DNA replication and must be periodically renewed. Over time, due to random mutation, the two gene copies will decrease in sequence similarity. Eventually, the silencer and silenced gene copies will diverge and the RdDM interaction will fail. Effective silencing could be maintained in the short-term if siRNAs from different silencing alleles provide the advantageous silencing at alternate methylation target sites. Ultimately, however, the compatibility between silencing and silenced copy will be broken. At this point, if siRNA silencing is favorable, selection would promote retention of a new gene duplicate performing the same function. Regulation of gene expression is a finely tuned process, and imprecise gene activation or inactivation can have significant fitness consequences. With tens of thousands of genes in plant genomes, regulatory mechanisms that can independently inactivate or attenuate individual genes would be a highly effective way to control a complex system.

### The biased duplication biased mutation (BDBM) model

**Fig 8:**
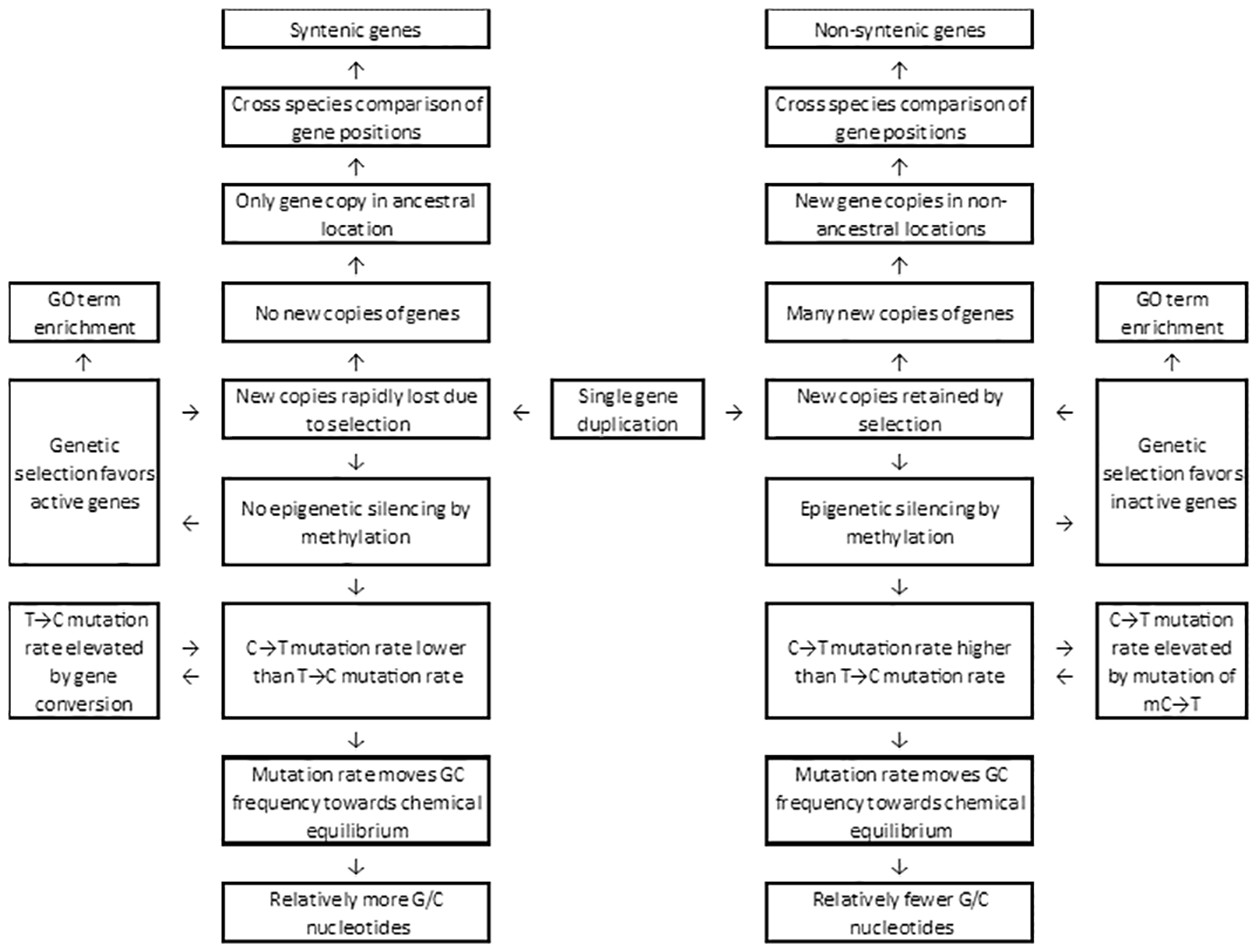
The biased gene duplication/biased mutation (BDBM) model.

We propose a model in which sequence duplication leads to siRNA production, which induces RdDM (Fig 8). Cytosine methylation causes biased local mutation rates favoring A/T nucleotides by relatively frequent mC→T mutations and/or by local reduction in gene conversion events that favor T→C substitutions. Methylation levels appear to be strongly associated with copy numbers of very similar sequences (Table 2), but many recent gene duplicates are too young for the slow mutational process to create a strong GC frequency bias. The observed methylation levels were highly heterogeneous, especially at CHH sites—however, we tacitly assume current methylation tendencies to be at least somewhat representative of past ones. Because the mC→T mutation rate is low, substantial differences in GC content could only arise if hyper-methylation is more likely in some sequences over millions of years. A strong correlation between GC3-50 and synteny would require repeated duplications of a subset of genes, with a different subset being less prone to duplication (or duplicate retention) and methylation. This mechanism, biased gene duplication and biased mutation (BDBM) could explain the observed relationship between local GC content and synteny.

A primary implication of BDBM in plants is that many gene duplications may be initially favored as “silencers” or “attenuators” of expression of the primary gene copy, rather than as a source of increased protein production or genetic redundancy. In many cases, retention of a silenced or attenuated gene copy might be favored over permanent loss of a conditionally favorable gene. As noted above, silenced gene copies might also be re-activated in certain environments, tissues or growth stages; for example, the reduced methylation associated with plant responses to many biotic or abiotic stresses [44, 46]. Stress induced demethylation can result from deactivation of the methylation replication mechanism or expression of active demethylases [43]. While temporary silencing of many genes might increase fitness, the silencing of other genes could be unfavorable. For example, gene duplication resulting in siRNA production and reduction of essential gene expression below a necessary threshold level would be selected against, and the siRNA producing copy would be purged from the population.

If a significant fraction of newly duplicated gene copies did result in siRNA production in plants, the result would be to divide genes into a spectrum of functional classes depending on the fitness consequences of silencing, reflecting the frequency of past duplication and methylation states producing GC nucleotide bias. At one end of the spectrum would be genes for which silencing confers reduced fitness; such genes would show little or no impact of methylation on nucleotide composition. Only duplicate copies that do not produce siRNAs would survive. In contrast, for genes in which the silenced state is usually favorable, recurrent duplications would be retained, thereby causing methylation and mutational bias. Genes would fall into two classes depending on favorability of siRNA silencing and given sufficient time, the mutational bias due to recurrent Cytosine methylation would produce GC content differences between the classes.

In mammals, local GC content variation is mostly due to variation in CG dinucleotide sequence frequencies [47]. This suggests that cytosine methylation is a principal factor influencing local GC content as most mammalian methylation is confined to CG sites. In plants, where methylation occurs in CG, CHG, and CHH contexts, differences in CG sequence frequency does not dominate local GC content, matching the methylation specificity, or possibly increased relative contribution of gene conversion.

There are other mechanisms and pathways that induce methylation in plants besides the siRNA pathway. However, if the other pathways are not correlated with gene duplication or synteny, they would not interfere with creation of a correlation between GC content and synteny resulting from a RdDM methylation events.

### Gene conversion

Another mechanism that could explain at least part of the GC correlation to synteny is gene conversion, which can produce a synergistic effect along with methylation. In plant genomes, presence/absence variation of genes is common (e.g., [48–50]. In genes that are variably present/absent within a species, some individuals will be heterozygous for the presence of the gene, and in those cases gene conversion cannot occur, as a syntenic copy for gene conversion is absent in meiosis. While it may still be possible for non-syntenic gene copies to be involved in gene conversion, since the process is linked to recombination, the non-syntenic rate of gene conversion would be expected to be substantially lower.

Gene conversion also is not technically a *de novo* mutation to a sequence, as it relies on an alternative gene copy to act as a template. For this reason, gene conversion acts more as a driver of allele frequency acting on an existing SNP in a population. The raw variation at synonymous sites in gene copies that have existed at the same location for millions of years will accumulate, providing the variation on which gene conversion can act to drive nucleotide frequencies following duplication. Genes that are syntenic across distantly related genomes are unlikely to contribute substantially to presence/absence variation, as they are highly stable in their genomic location.

On the other hand, recently duplicated genes at a new locus will be initially present as rare variants in a population and thus unlikely to be homozygous present, as would be required for gene conversion to be a major factor. Moreover, newly duplicated genes may not have accumulated the polymorphisms necessary for gene conversion to occur; as noted above, gene conversion does not create new mutations as much as it drives GC content at already polymorphic loci. Genes that are frequently duplicated and have moved around the genome over time undergo a period of reduced gene conversion after each duplication event, which will reduce the frequency of T→C changes. Over time this could result in a biased nucleotide content, especially for genes that have undergone repeated duplications.

### Alternative explanations and a role for transposons

An alternate hypothesis might be that correlation between GC3-50 and synteny in the grasses is due to an unknown process that preferentially duplicates methylated sequences or low GC content genes. An argument against such a mechanism is that transposable element activity can be enhanced by disrupting their normal hyper-methylated state—indeed, transposon activity is known to increase when sequences are hypo-methylated [51, 52]. As many gene duplications in plants are related to transposon activity [53, 54], it is difficult to justify an explanation that depends on preferential duplication of hyper-methylated sequences. More likely, the transposable elements are a mechanism that produce a situation in which hypo-methylated genes are regularly duplicated and the fitness effects of an extra, potentially siRNA-producing copy are repeatedly tested. Alternatively, transposons may restore methylation that has declined due to decay of associated siRNA producing gene copies. In this view, unfavorable duplicated sequences would be purged from the population by selection.

The tendency of DNA transposons to carry genes or gene fragments may periodically renew siRNA-based regulation for many genes [55]. The siRNA-producing silencer copy in any individual would be under less immediate selective pressure than protein coding genes, as the silencer copy is only needed to re-establish methylation after it has been lost. Therefore, a silencer gene copy may only be needed to re-silence its target occasionally. Occasionally used silencer gene copies would be expected to gradually decline in effectiveness due to limited immediate selection against loss of function mutations, until they can no longer re-initiate methylation at the target. At this point, a newly duplicated siRNA-producing silencer copy would be advantageous. Such a system would favor the ongoing duplication of certain genes to refresh siRNA silencing. This raises the intriguing but speculative hypothesis that the need to re-establish silencing may favor retention of mechanisms that can duplicate arbitrary genomic segments and integrate the new copies back into the genome. Such a mechanism may be a feature of several families of plant transposable elements such as Pack-MULEs [53], helitrons [54, 56], or CACTA elements [57]. The restoration and creation of favorable methylation linked gene copy pairs of silencer and silenced copy, might be a reason why transposable element systems capable of duplicating genes have survived.

### Effects on different classes of genes

A class of genes that in which duplicated copies are lost soon after gene duplication due to selection, has been recognized previously following allopolyploid whole genome duplication (WGD) events. Such WGD events have been associated with increases in DNA methylation level [58], changes in gene expression [59], and the rapid loss of one copy for certain classes of genes [60, 61]. Essential genes that are silenced by methylation following a WGD event would confer strong selective pressure favoring removal of the ‘extra,’ methylation-initiating copy. The elevated mutation rate of mC following polyploidy could also accelerate sequence divergence leading to divergence in gene function. Hypo-methylated sequences could also be targeted for rapid removal by DNA elimination processes that normally remove heterochromatic transposons [62]. The accelerated loss of heterochromatic hypermethylated sequences might also facilitate gene movement after single gene duplications involving RdDM.

GO term enrichment suggests that many classes of stress response or pathogen response genes are enriched in the highly duplicated, non-syntenic group, consistent with prior studies indicating high mobility of these gene classes [63, 64]. Defense-related GO terms are also over-represented amongst genes enriched in CHG and CHH methylation across species [65], consistent with frequent silencing of these genes. These two lines of evidence suggest that defense-related genes often occur in silenced dormant status in the genome, possibly being activated by stress-induced demethylation when needed. This could help explain why GO terms such as “defense response,” “hypersensitive response,” “detection of bacterium,” and “response to biotic stimulus” were enriched for non-syntenic genes in all nine species tested. Some other classes of stress response genes such as “cellular response to water deprivation,” “cellular response to salt stress,” or “defense response to fungus” showed family expansion with very strong enrichment in the non-syntenic class for some species, while being maintained in very small numbers in other species. Pathogen-related defense genes might be prone to duplication as new copies can adapt to new strains while maintaining the original copy to preserve resistance to existing strains. Conversely, many of the GO terms that showed enrichment in the low copy, syntenic class of genes involve functions or structures that are constitutively required. These potential housekeeping genes, which include several different GO classifications that contained the words “nucleus,” “Golgi,” “chloroplast,” and “transcription” in their description, were enriched in the syntenic fraction for all nine species.

It is worth noting here that active demethylation can be initiated in specific tissues or developmental stages, potentially allowing for the re-activation of genes needed to produce various developmental structures or cell types [66, 67]. Some non-germ line tissues have been associated with targeted demethylation such as roots [68], egg companion cells [69], and pollen vegetative nuclei [70]. Therefore, some tissue-specific genes might also be enriched in the non-syntenic, lower GC3-50 subset. For example, proteins associated with “recognition of pollen” were overrepresented in the non-syntenic fraction for all nine species tested, and with the pattern being highly significant (i.e., *P* < 0.001) for eight of the species. This finding is consistent with an observation that many pollen-specific genes are enriched for methylation [71].

### Implications for population genetics and selection

Individuals with specific siRNAs can pass gene-specific silencing on to progeny that do not inherit the siRNA-encoding sequence itself, via methylation replication at CG and CHG sites. Therefore, methylation initially induced by siRNA in an individual may correspond to the sequences encoding siRNAs not present in its own genome [72]. In an outcrossing species, individuals may possess some favorable siRNAs but lack others. With interbreeding and the maintenance of methylation, an individual could have many methylation-silenced genes that were induced in different ancestral individuals, even when it currently lacks the siRNA producing capacity. This multi-generational trade in siRNAs could provide fitness advantages to a local population, depending on the siRNA diversity within that population. The trade in epigenetic signals might also contribute to hybrid vigor [73, 74].

The frequency of siRNA-producing sequences for some genes might be under balancing selection since gene silencing providing episodic advantages during periods of environmental stress or pathogen outbreaks. At the population level, cytosine methylation also provides plants with a degree of adaptability or acclimation, allowing them to respond to local changes in conditions or different environments. Examination of many different accessions of *Arabidopsis*, for example, showed variation in methylation status [75] presumably correlated with optimization to local conditions. A gene for which silencing may be favorable under normal conditions may provide a fitness advantage when unsilenced under periodic stresses such as drought or disease. Natural or stress-related loss of methylation might result in a plant producing a range of differentially methylated seeds, perhaps increasing the chance that at least some of its offspring have an optimal mix of silenced and unsilenced genes for a range of potential environmental conditions. Similarly, crossing with individuals producing advantageous siRNAs will return genes to silenced status. If an interbreeding population contains a degree of heterozygosity for the specific siRNAs that could silence a gene, each generation could have a fraction of the population with unsilenced vs. silenced gene copies. Because of the silencing effect of the siRNAs in heterozygous individuals, the fraction of the population with silenced gene copies changes much more rapidly than the frequencies of the initiating siRNAs could change due to selection. This mechanism would offer better adaptability and robustness towards shifting environment stress.

### Implications for the molecular clock

The BDBM theory and biased gene conversion rates has implications for the use of DNA sequence variation as a ‘molecular clock,’ a statistical approach for estimating the age of past evolutionary events [44, 76]. This approach assumes that mutations occur at random in a clock-like manner within a genome. By approximating the number of mutational changes that have occurred between two sequences while accounting for multiple substitutions, one can estimate how much time has passed since the sequences shared a common ancestor. The BDBM theory suggests that frequently methylated genes will undergo C→T mutations at a more rapid rate than hypomethylated genes, causing molecular clock calculations to overestimate the ages of methylated gene duplicates. The BDBM theory would also predict over estimation of ages for within-genome gene duplications or polyploidy events compared to similarly timed speciation events. However, gene conversion rates will also be reduced for newly duplicated genes that frequently occur as present/absent heterozygotes, meaning that apparent G→A mutation rates could be slowed due to reduction in gene conversion amongst newly duplicated genes. Different clock rates could thus be applicable to genes with different rates of duplication or historical methylation states. Since whole genome duplication events are often associated with temporary increases in the silencing of duplicate gene copies, a temporary acceleration of mutational rates might make a polyploidy event appear older than it actually is, resulting in incorrect phylogenetic placement [77]. Ideally, molecular clock comparisons should strive to use genes with similar duplication histories to calibrate mutation rates and estimate dates.

## Conclusion

The potential retention of duplicate gene sequences due to their role in the establishment (or re-establishment) of siRNA-induced silencing of the progenitor gene changes the traditional paradigm of duplicate gene evolution for plants. Instead of being a lingering feature of pseudogenes [78], siRNAs might confer a selective advantage to the creation and retention of gene copies that produce them. The traditional view that the primary advantage of gene duplication is initially increased protein production, possibly followed by eventual sub- or neo-functionalization leading to functional innovation, could by expanded if some new gene copies have immediately advantageous effects as epigenetic regulators. If the silencing copy retains (or regains) expression as a protein coding gene, the maintenance of a redundant gene copy experiencing selection for advantageous siRNA production could also provide raw material for evolutionary experimentation as a variant protein. Many gene duplications do not result in RdDM, and the duplicate copies may be retained for traditional reasons such as additional gene product, genetic redundancy, or benefits associated with sub/neo-functionalization. This would help to explain the limited correlations between methylation, duplication, and GC3 content seen for some species, especially the eudicots. The BDBM model does not purport to be an exclusive pathway leading to methylation as other mechanisms can give rise to *de novo* methylation notably CMT2 [79]. The BDBM process could both favor retention of duplicate genes, which are the source of most new genes [80], and alter the mutational spectrum of duplicated genes.

## Materials and methods

### Genomes, genes and synteny

Genome assemblies and predicted gene sets were obtained through the SynMap portal at CoGe (https://genomevolution.orG/Coge/SynMap.pl; [81]. The genome versions used are listed in S2 Table in S5 File. Detection of synteny was performed using SynMap at CoGe based on relative gene order and BLAST using tblastx and a minimum syntenic block size of 7 gene pairs [82]. Synteny status was merged for all splice variants of a gene and the coding sequence for the first splice variant was used in downstream analysis. Within plant genes, GC3 is also correlated with distance from the start of the coding sequence (S5 File) [16], in grass genes the GC3 content is most variable at the beginning of genes. Genes shorter than 50 codons, genes with unknown bases, and predicted genes not starting with ATG were not used for analysis. Composition at the 3rd position of the first 50 codons was calculated with Microsoft Excel 2013 and relationships and correlations were calculated in Excel or JMP Pro v13.0 (SAS Institute, Inc., Cary, NC). The terms syntenic and non-syntenic were used instead of paralog and ortholog to reflect positional considerations. Syntenic genes include orthologs in different species that are in corresponding locations, as well as paralogs duplicated by polyploidy events within a species that remain in corresponding locations.

### GO terms

Gene ontology (GO) analyses were performed by comparing gene sequences to the manually annotated Swiss-Prot database using BLASTP with *E* < 10^−10^ and transferring all associated GO terms of the best match to the query sequence. Within-genome gene copy number was assessed by using BLASTN of the first 150 base pairs of each gene to the whole genome assembly at high stringency (E < 10^−30^ corresponding to > 90% identity in most cases) and counting the number of significant hits.

### Methylome analysis

Previously published whole-genome bisulfite sequencing data [65, 83–85] was downloaded from the Short-Read Archive (S2 Table in S5 File) and remapped to each species’ respective genome using methylpy [71]. Bisulfite sequencing used leaves, except for maize where unfertilized outer ears were used. Custom python scripts and pybedtools [86] were used to map methylation data to the first 150 base pairs of each transcript and call the weighted methylation level [87] (S1 and S2 Files). All methylation analysis scripts, and tables of methylation levels are available on Github (https://github.com/niederhuth/Bowers-Gene-Duplication-Methylation).

## Supporting information

S1 File Gene list showing synteny, methylation and GC content for 5 dicot species. (XLSX)

S2 File Gene list showing synteny, methylation and GC content for 4 monocot species. (XLSX)

S3 File GenBank information and annotation versions of genomes used in this study. (XLSX)

S4 File Gene Ontology (GO) terms associated with syntenic and non-syntenic genes. (XLSX)

S5 File Figures showing dot plots of synteny, GC3 content for syntenic and non-syntenic genes, and copy number vs GC3 content for all 9 species. (DOCX)
