# Supplementary material for: GC content of plant genes is linked to past gene duplications
Source: PLoS One. 2022 Jan 13;17(1):e0261748. doi: 10.1371/journal.pone.0261748 (PMC8758071; doi:10.1371/journal.pone.0261748)
Supplement: S5 File — (DOCX) [file pone.0261748.s005.docx]

**Supplemental Figure 1**

*Arabdopsis thaliana* to *Arabidopsis thaliana* synteny. Dots indicate syntenic gene pairs conserved in gene order between the two species. Syntenic segments were identified as stretches of seven or more genes conserved in order between the two genomes. The x- axis represents 26,725 AT genes and the y-axis represents 26,725 AT genes.

**Supplemental Figure 2**

*Arabidopsis thaliana* to *Prunus persica* synteny. Dots indicate syntenic gene pairs conserved in gene order between the two species. Syntenic segments were identified as stretches of seven or more genes conserved in order between the two genomes. The x-axis represents 26,725 AT genes and the y-axis represents 24,702 PP genes.

**Supplemental Figure 3**

Arabidopsis thaliana to *Populus tricocarpa* synteny. Dots indicate syntenic gene pairs conserved in gene order between the two species. Syntenic segments were identified as stretches of seven or more genes conserved in order between the two genomes. The x-axis represents 26,725 AT genes and the y-axis represents 43,415 PT genes.

**Supplemental Figure 4**

Arabidopsis thaliana to *Solanum lycopersicum* synteny. Dots indicate syntenic gene pairs conserved in gene order between the two species. Syntenic segments were identified as stretches of seven or more genes conserved in order between the two genomes. The x-axis represents 26,725 AT genes and the y-axis represents 21,555 SL genes.

**Supplemental Figure 5**

*Arabidopsis thaliana* to *Vitis vinifera* synteny. Dots indicate syntenic gene pairs conserved in gene order between the two species. Syntenic segments were identified as stretches of seven or more genes conserved in order between the two genomes. The x-axis represents 26,725 AT genes and the y-axis represents 22,793 VV genes.

**Supplemental Figure 6**

*Prunus persica* to *Prunus persica* synteny. Dots indicate syntenic gene pairs conserved in gene order between the two species. Syntenic segments were identified as stretches of seven or more genes conserved in order between the two genomes. The x-axis represents 24,702 PP genes and the y-axis represents 24,702 PP genes.

**Supplemental Figure 7**

*Prunus persica* to *Populus tricocarpa* synteny. Dots indicate syntenic gene pairs conserved in gene order between the two species. Syntenic segments were identified as stretches of seven or more genes conserved in order between the two genomes. The x-axis represents 24,702 PP genes and the y-axis represents 43,415 PT genes.

**Supplemental Figure 8**

*Prunus persica* to *Solanum lycopersicum* synteny. Dots indicate syntenic gene pairs conserved in gene order between the two species. Syntenic segments were identified as stretches of seven or more genes conserved in order between the two genomes. The x-axis represents 24,702 PP genes and the y-axis represents 21,555 SL genes.

**Supplemental Figure 9**

*Prunus persica* to *Vitis vinifera* synteny. Dots indicate syntenic gene pairs conserved in gene order between the two species. Syntenic segments were identified as stretches of seven or more genes conserved in order between the two genomes. The x-axis represents 24,702 VV genes and the y-axis represents 22,293 VV genes.

**Supplemental Figure 10**

*Populus tricocarpa* to *Populus tricocarpa* synteny. Dots indicate syntenic gene pairs conserved in gene order between the two species. Syntenic segments were identified as stretches of seven or more genes conserved in order between the two genomes. The x-axis represents 43,415 PT genes and the y-axis represents 43,415 PT genes.

**Supplemental Figure 11**

*Populus tricocarpa* to *Solanum lycopersicum* synteny. Dots indicate syntenic gene pairs conserved in gene order between the two species. Syntenic segments were identified as stretches of seven or more genes conserved in order between the two genomes. The x-axis represents 43,415 PT genes and the y-axis represents 21,555 SL genes.

**Supplemental Figure 12**

*Populus tricocarpa* to *Vitis vinifera* synteny. Dots indicate syntenic gene pairs conserved in gene order between the two species. Syntenic segments were identified as stretches of seven or more genes conserved in order between the two genomes. The x-axis represents 43,415 PT genes and the y-axis represents 22,293 VV genes.

**Supplemental Figure 13**

*Solanum lycopersicum* to *Solanum lycopersicum* synteny. Dots indicate syntenic gene pairs conserved in gene order between the two species. Syntenic segments were identified as stretches of seven or more genes conserved in order between the two genomes. The x-axis represents 21,555 SL genes and the y-axis represents 21,555 SL genes.

**Supplemental Figure 14**

*Solanum lycopersicum* to *Vitis vinifera* synteny. Dots indicate syntenic gene pairs conserved in gene order between the two species. Syntenic segments were identified as stretches of seven or more genes conserved in order between the two genomes. The x-axis represents 21,555 SL genes and the y-axis represents 22,293 VV genes.

**Supplemental Figure 15**

*Vitis vinifera* to *Vitis vinifera* synteny. Dots indicate syntenic gene pairs conserved in gene order between the two species. Syntenic segments were identified as stretches of seven or more genes conserved in order between the two genomes. The x-axis represents 22,293 VV genes and the y-axis represents 22,293 VV genes.

**Supplemental Figure 16**

*Brachypodium distachyon* to *Brachypodium distachyon* synteny. Dots indicate syntenic gene pairs conserved in gene order between the two species. Syntenic segments were identified as stretches of seven or more genes conserved in order between the two genomes. The x-axis represents 31,545 BD genes and the y-axis represents 31,545 BD genes.

**Supplemental Figure 17**

*Brachypodium distachyon* to *Oryza sativa* synteny. Dots indicate syntenic gene pairs conserved in gene order between the two species. Syntenic segments were identified as stretches of seven or more genes conserved in order between the two genomes. The x-axis represents 31,545 BD genes and the y-axis represents 41,960 OS genes.

**Supplemental Figure 18**

*Brachypodium distachyon* to *Sorghum bicolor* synteny. Dots indicate syntenic gene pairs conserved in gene order between the two species. Syntenic segments were identified as stretches of seven or more genes conserved in order between the two genomes. The x-axis represents 31,545 BD genes and the y-axis represents 33,810 SB genes.

**Supplemental Figure 19**

*Brachypodium distachyon* to *Zea mays* synteny. Dots indicate syntenic gene pairs conserved in gene order between the two species. Syntenic segments were identified as stretches of seven or more genes conserved in order between the two genomes. The x-axis represents 31,545 BD genes and the y-axis represents 38,564 ZM genes.

**Supplemental Figure 20**

*Oryza sativa* to *Oryza sativa* synteny. Dots indicate syntenic gene pairs conserved in gene order between the two species. Syntenic segments were identified as stretches of seven or more genes conserved in order between the two genomes. The x-axis represents 41,960 OS genes and the y-axis represents 41,960 OS genes.

**Supplemental Figure 21**

*Oryza sativa* to *Zea mays* synteny. Dots indicate syntenic gene pairs conserved in gene order between the two species. Syntenic segments were identified as stretches of seven or more genes conserved in order between the two genomes. The x-axis represents 41,960 OS genes and the y-axis represents 38,564 ZM genes.

**Supplemental Figure 22**

*Sorghum bicolor* to *Sorghum bicolor* synteny. Dots indicate syntenic gene pairs conserved in gene order between the two species. Syntenic segments were identified as stretches of seven or more genes conserved in order between the two genomes. The x-axis represents 33,810 SB genes and the y-axis represents 33,810 SB genes.

**Supplemental Figure 23**

*Sorghum bicolor* to *Zea mays synteny*. Dots indicate syntenic gene pairs conserved in gene order between the two species. Syntenic segments were identified as stretches of seven or more genes conserved in order between the two genomes. The x-axis represents 33,810 SB genes and the y-axis represents 38,564 ZM genes.

**Supplemental Figure 24**

*Zea mays* to *Zea mays* synteny. Dots indicate syntenic gene pairs conserved in gene order between the two species. Syntenic segments were identified as stretches of seven or more genes conserved in order between the two genomes. The x-axis represents 38,564 ZM genes and the y-axis represents 38,564 ZM genes.


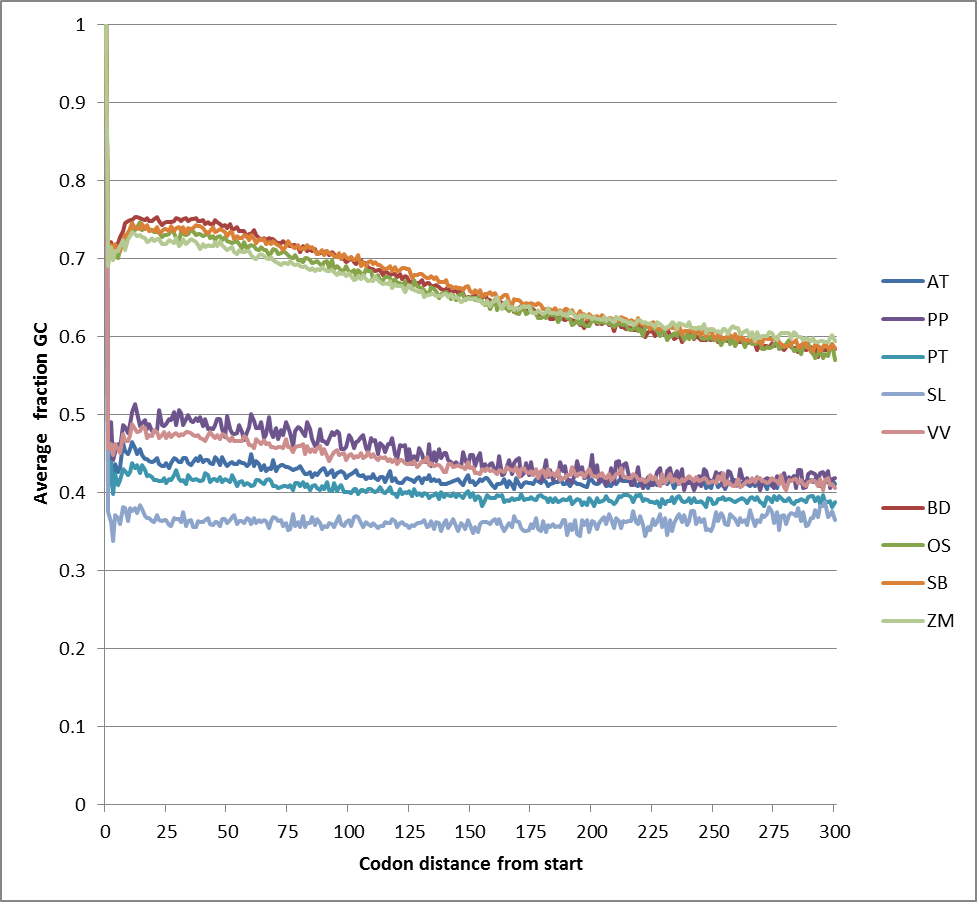


**Supplemental Figure 25**

Average fraction GC nucleotides for 3rd bp of codons by distance from the start for nine plant species.


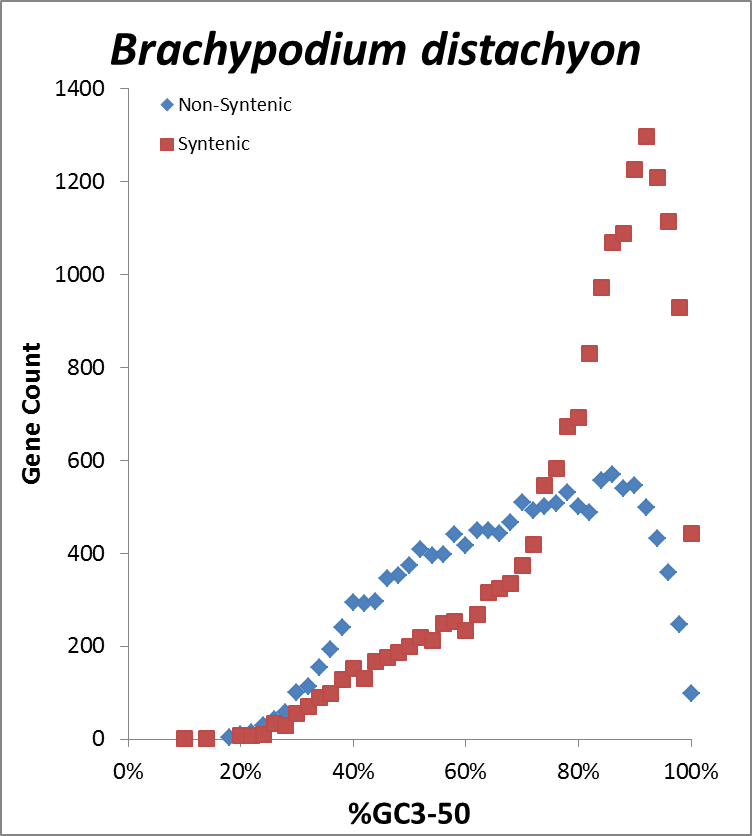


**Supplemental Figure 26**

Synteny status of genes by percent GC content at the third position for the first 50 codons (%GC3-50) for17406 syntenic and 14139 non-syntenic *Brachypodium distachyon* genes.


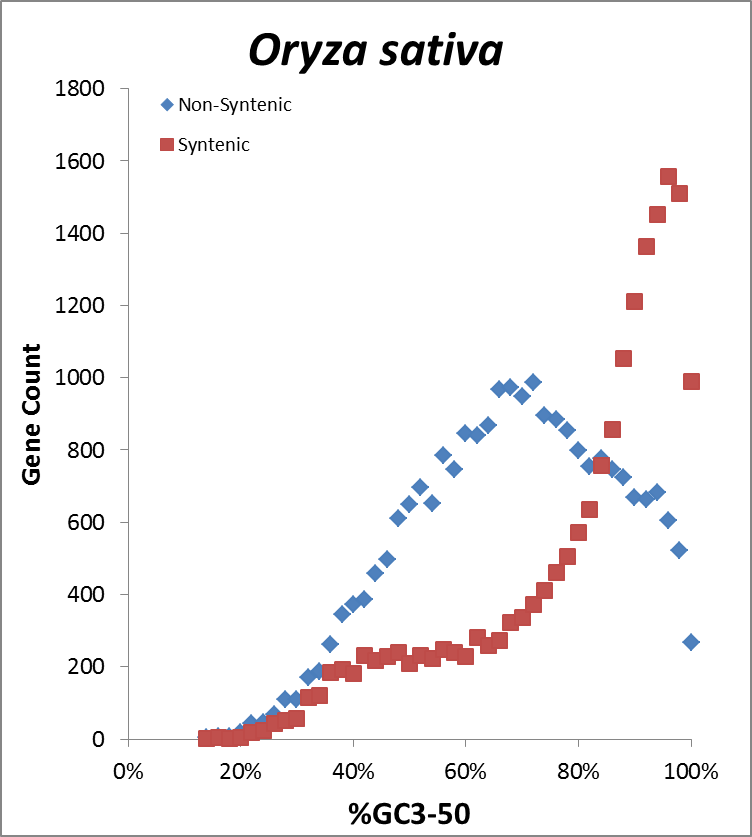


**Supplemental Figure 27**

Synteny status of genes by percent GC content at the third position for the first 50 codons (%GC3-50) for 18464 syntenic and 23496 non-syntenic *Oryza sativa* genes.


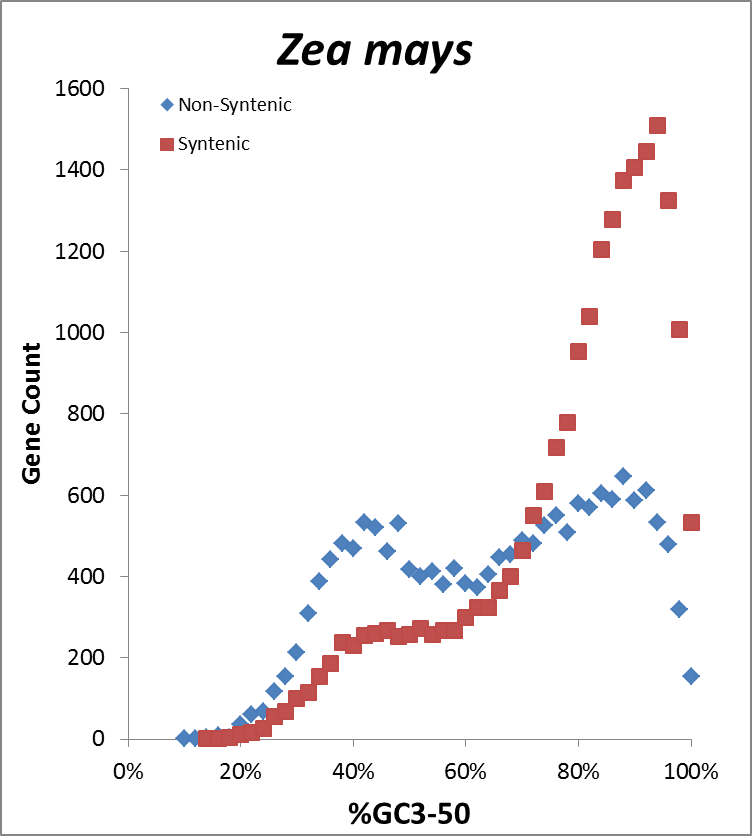


**Supplemental Figure 28**

Synteny status of genes by percent GC content at the third position for the first 50 codons (%GC3-50) for21456 syntenic and 17108 non-syntenic *Zea mays* genes.


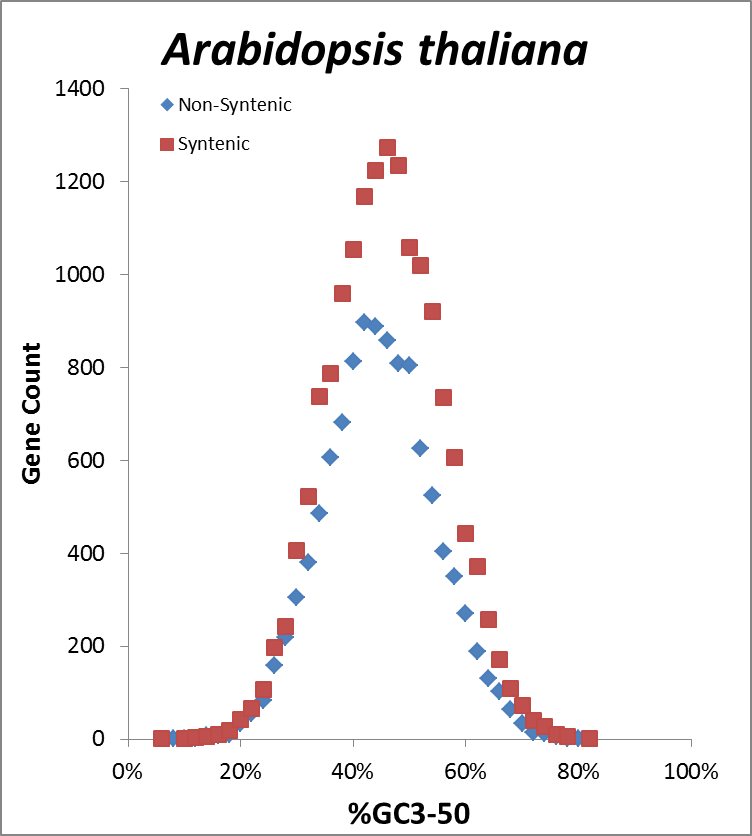


**Supplemental Figure 29**

Synteny status of genes by percent GC content at the third position for the first 50 codons (%GC3-50) for 15896 syntenic and 10829 non-syntenic *Arabidopsis thaliana* genes.


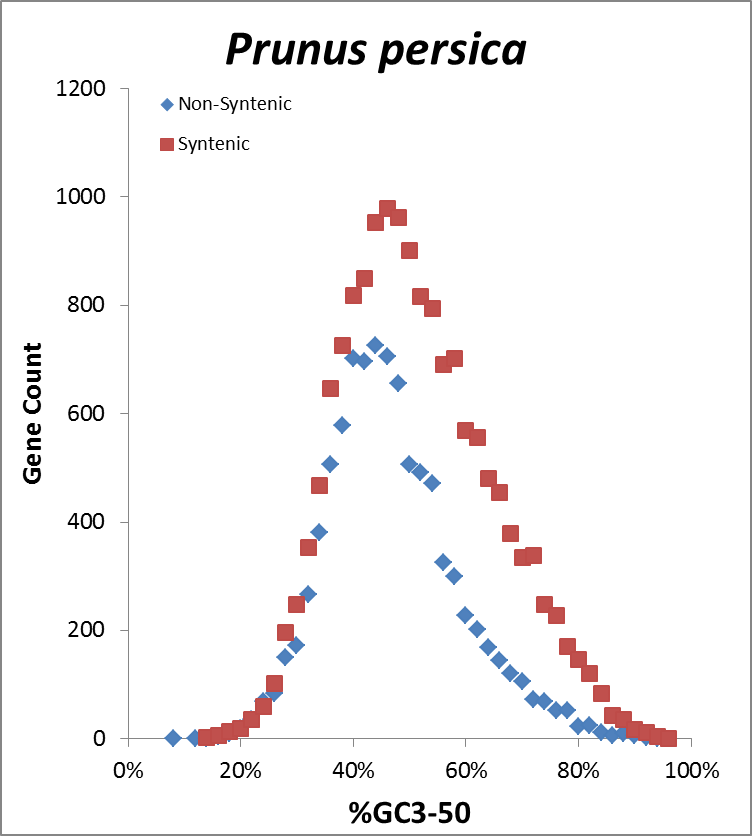


**Supplemental Figure30**

Synteny status of genes by percent GC content at the third position for the first 50 codons (%GC3-50) for 15551 syntenic and 9151 non-syntenic *Prunus persica* genes.


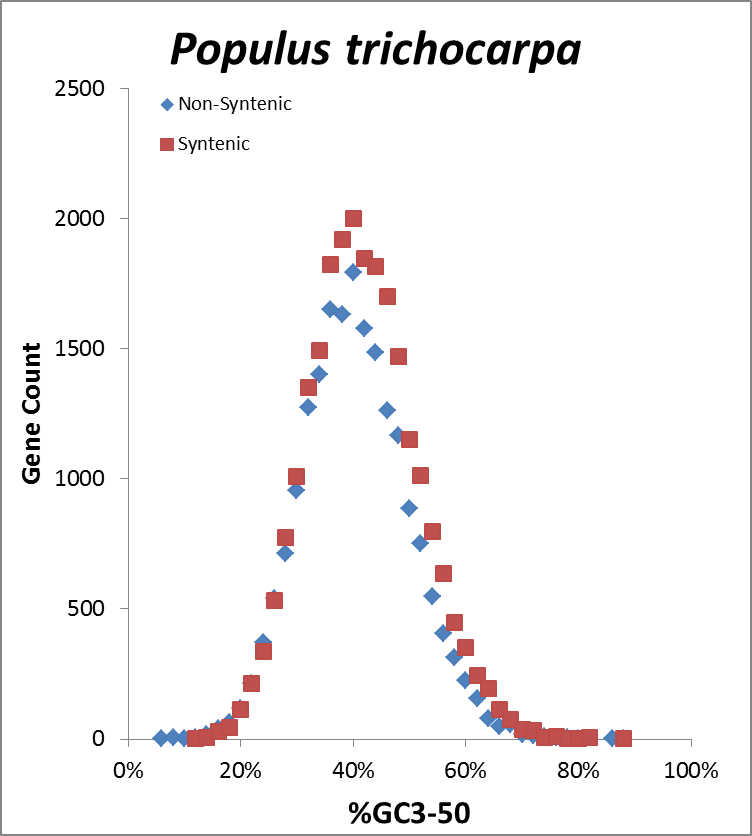


**Supplmental Figure 31**

Synteny status of genes by percent GC content at the third position for the first 50 codons (%GC3-50) for 23600 syntenic and 19815 non-syntenic *Populus tricocarpa* genes.


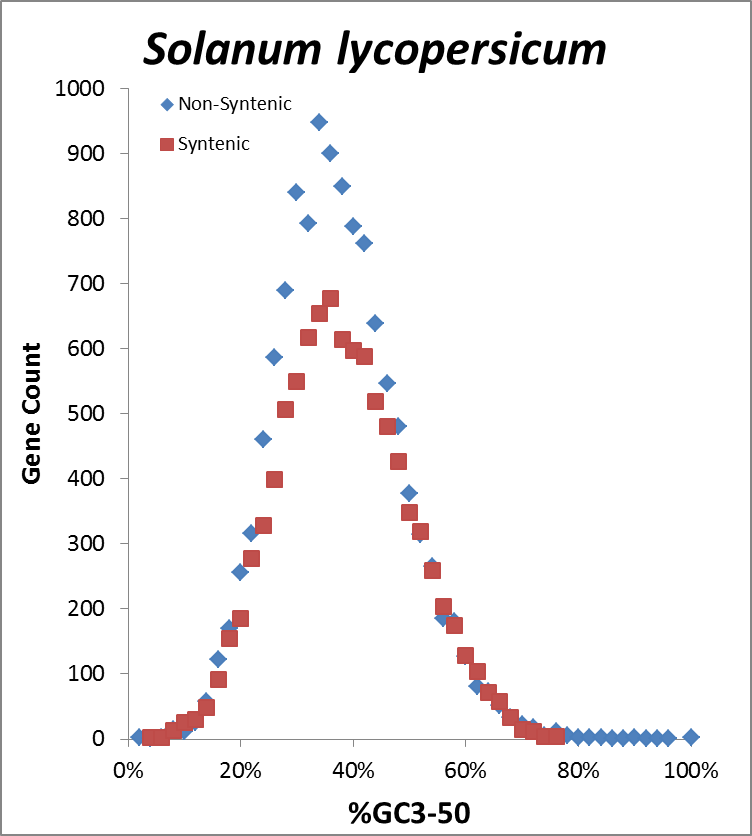


**Supplemental Figure 32**

Synteny status of genes by percent GC content at the third position for the first 50 codons (%GC3-50) for 9524 syntenic and 12031 non-syntenic *Solanum lycopersicum* genes.


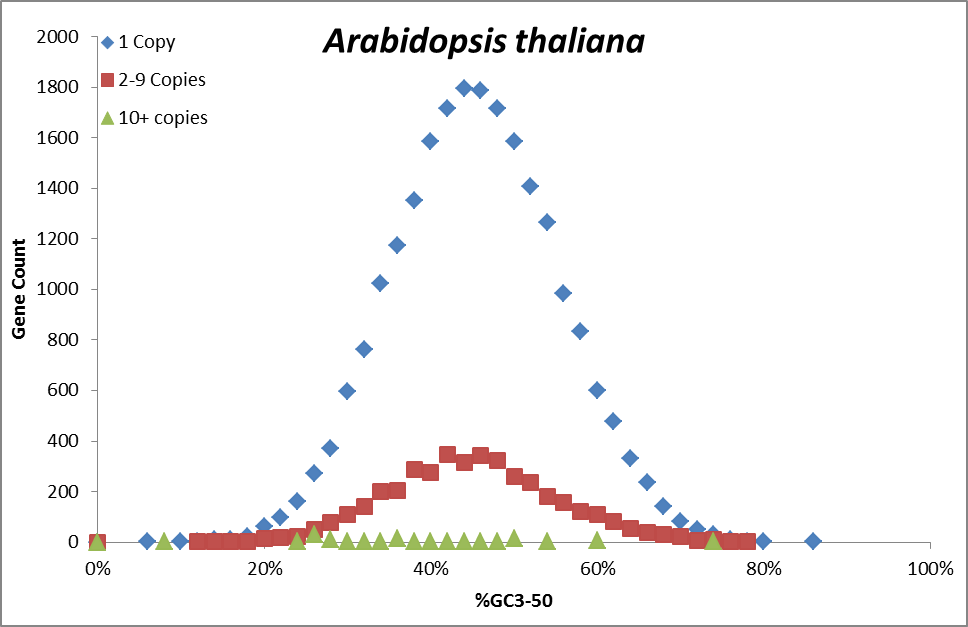


**Supplemental Figure 33**

Gene count distribution by %GC3-50 and copy number of gene within the genome assembly by number of high similarity BLAST hits for 22,565 single copy, 4,052 moderately repetitive and 108 repetitive *Arabidopsis thaliana* genes.


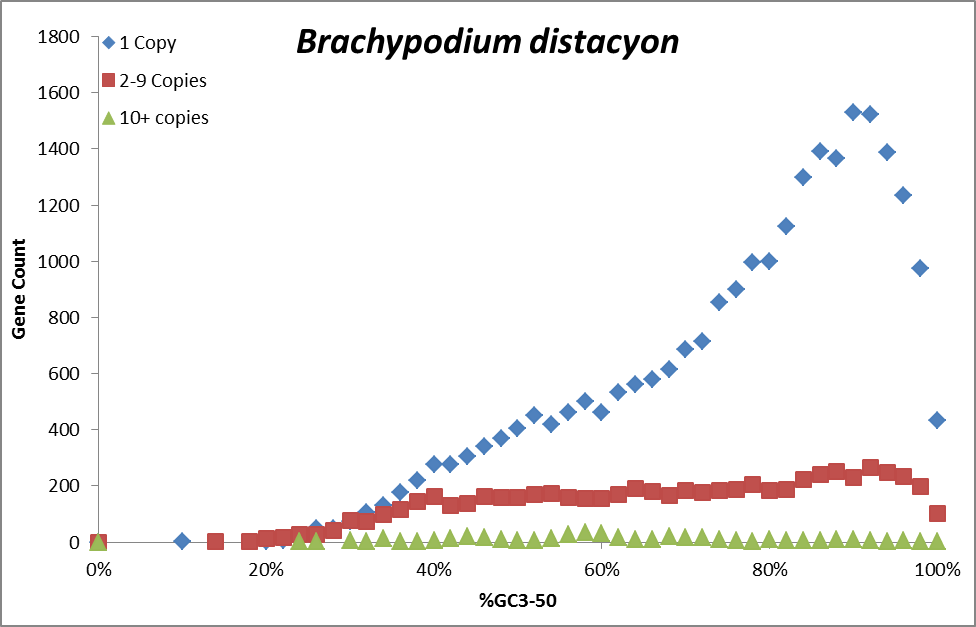


**Supplemental Figure 34**

Gene count distribution by %GC3-50 and copy number of gene within the genome assembly by number of high similarity BLAST hits for 247,90 single copy, 6,368 moderately repetitive and 387 repetitive *Brachypodium distacyon* genes.


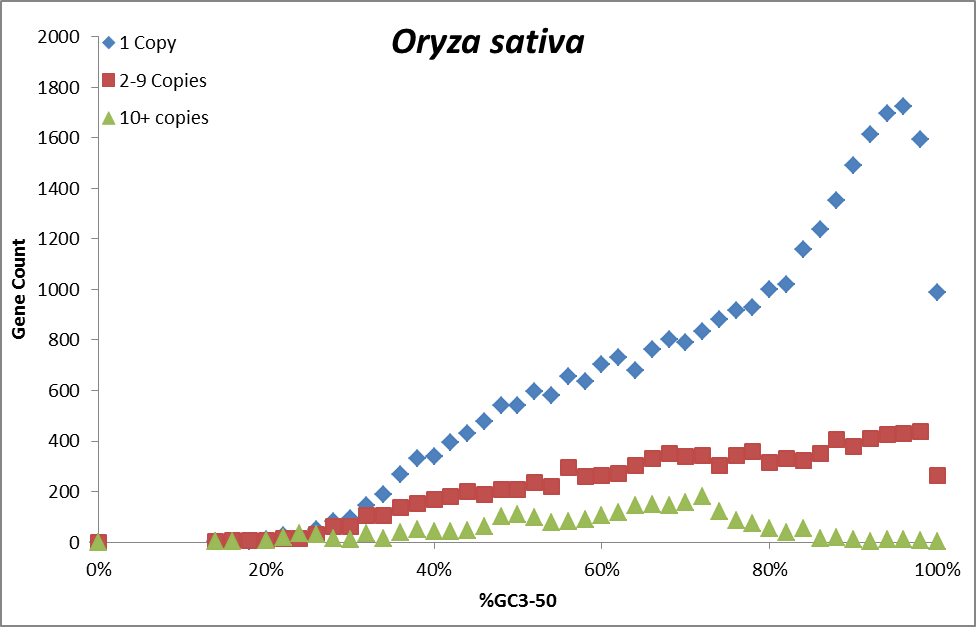


**Supplemental Figure 35**

Gene count distribution by %GC3-50 and copy number of gene within the genome assembly by number of high similarity BLAST hits for 29,299 single copy, 10,153 moderately repetitive and 2,508 repetitive *Oryza sativa* genes.


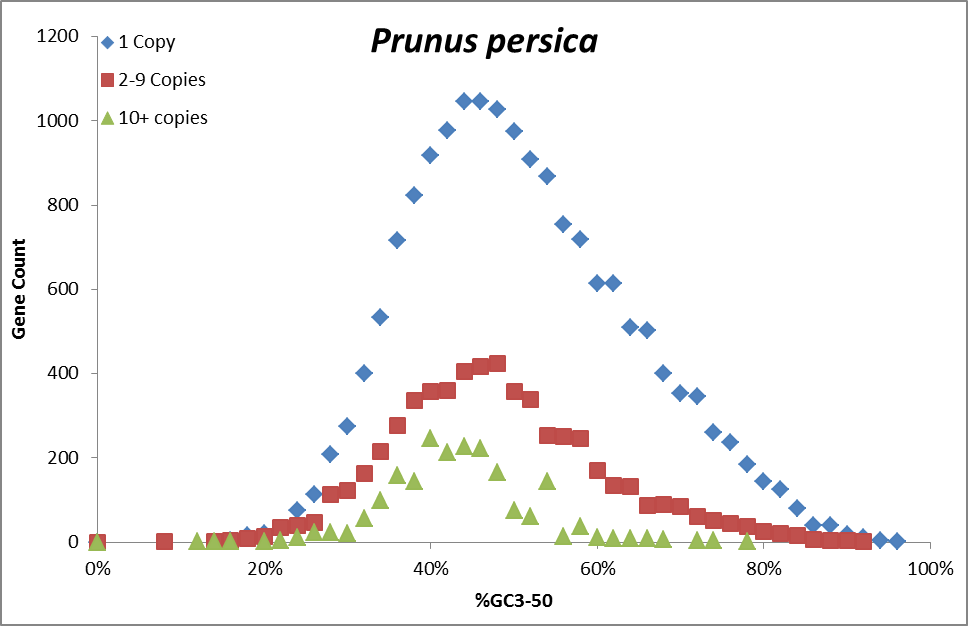


**Supplemental Figure 36**

Gene count distribution by %GC3-50 and copy number of gene within the genome assembly by number of high similarity BLAST hits for 16,939 single copy, 5,762 moderately repetitive and 2,001 repetitive *Prunus persica* genes.


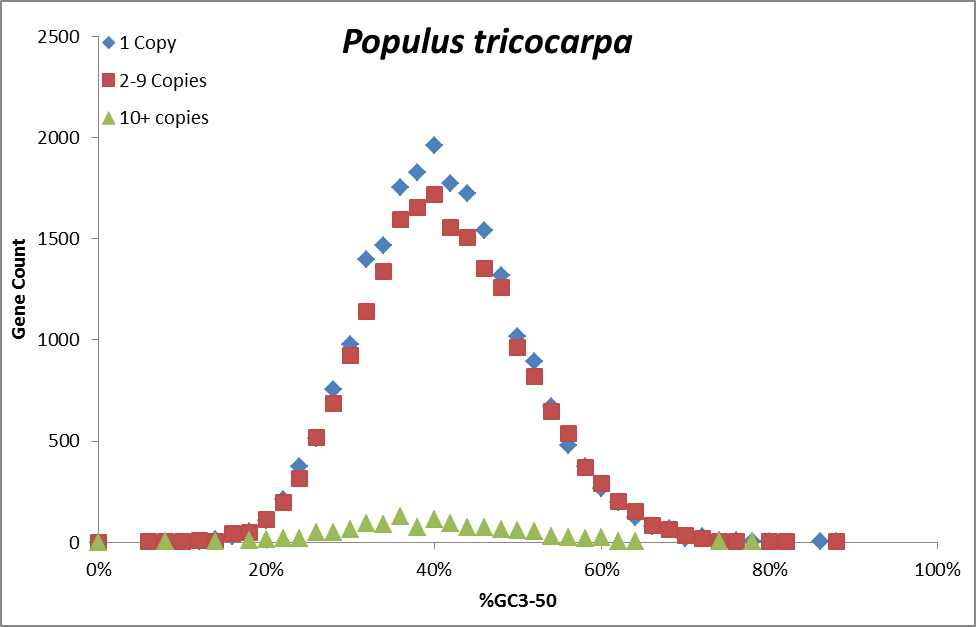


**Supplemental Figure 37**

Gene count distribution by %GC3-50 and copy number of gene within the genome assembly by number of high similarity BLAST hits for 22,023 single copy, 20172 moderately repetitive and 1220 repetitive *Populus tricocarpa* genes.


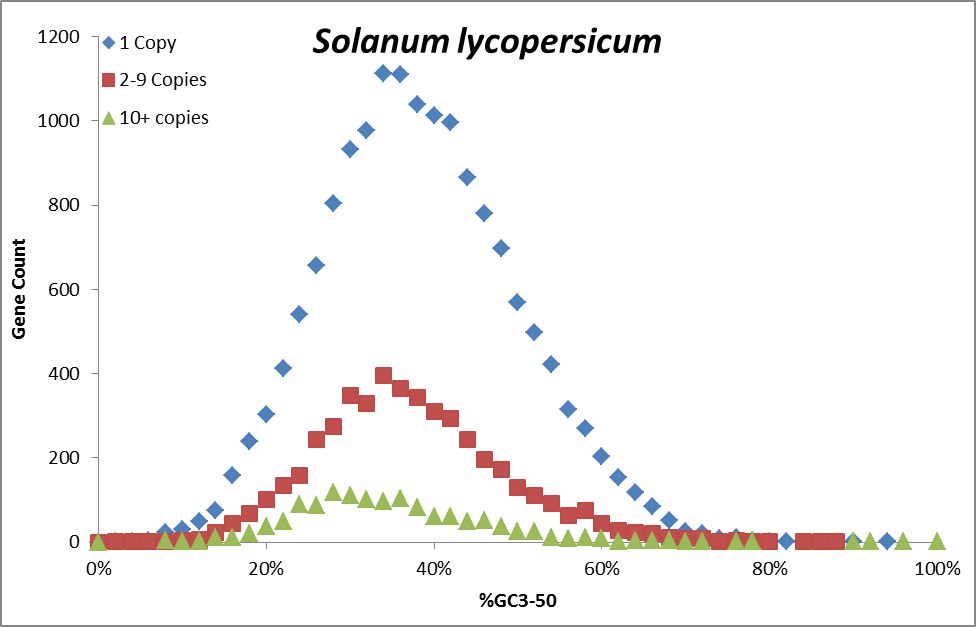


**Supplemental Figure 38**

Gene count distribution by %GC3-50 and copy number of gene within the genome assembly by number of high similarity BLAST hits for 15,575 single copy, 4,684 moderately repetitive and 1,296 repetitive *Solanum lycopersicum* genes.


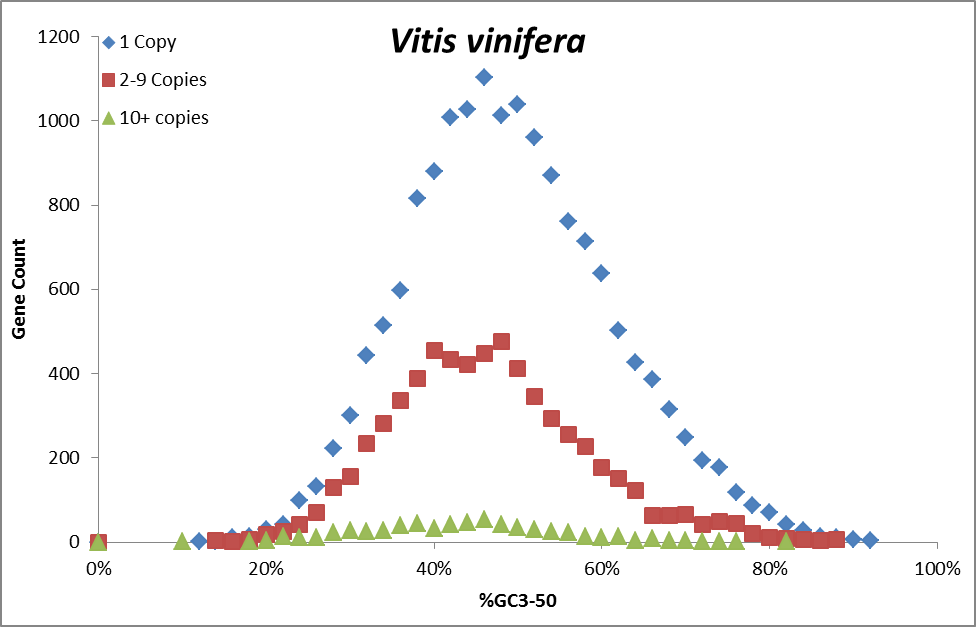


**Supplemental Figure 39**

Gene count distribution by %GC3-50 and copy number of gene within the genome assembly by number of high similarity BLAST hits for 15,868 single copy, 6,298 moderately repetitive and 627 repetitive *Vitis vinifer*a genes.


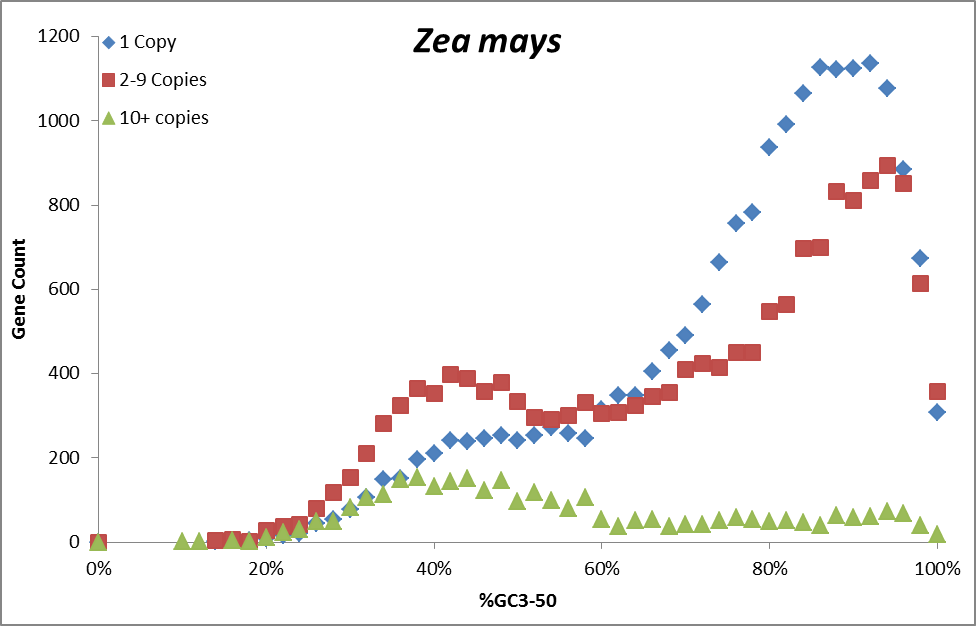


**Supplemental Figure 40**

Gene count distribution by %GC3-50 and copy number of gene within the genome assembly by number of high similarity BLAST hits for 18,866 single copy, 16,586 moderately repetitive and 3024 repetitive *Zea mays* genes.

|  |  | Non Syntenic | Syntenic |
| --- | --- | --- | --- |
| *Arabidopsis* | 1 copy | 8208 | 14357 |
| *thaliana* | 2-9 Copies | 2516 | 1536 |
|  | 10+ copies | 105 | 3 |
| *Prunus* | 1 copy | 4130 | 12809 |
| *persica* | 2-9 Copies | 7188 | 4336 |
|  | 10+ copies | 14270 | 5740 |
| *Populus* | 1 copy | 9634 | 12389 |
| *tricocarpa* | 2-9 Copies | 18092 | 22252 |
|  | 10+ copies | 11350 | 850 |
| *Solanum* | 1 copy | 7186 | 8660 |
| *lycopersicum* | 2-9 Copies | 4130 | 755 |
|  | 10+ copies | 1261 | 123 |
| *Vitis* | 1 copy | 9145 | 15645 |
| *vinifera* | 2-9 Copies | 9302 | 3434 |
|  | 10+ copies | 3430 | 440 |
| *Brachypodium* | 1 copy | 4371 | 11497 |
| *distachyon* | 2-9 Copies | 4306 | 1992 |
|  | 10+ copies | 572 | 55 |
| *Oryza* | 1 copy | 13616 | 15683 |
| *sativa* | 2-9 Copies | 15186 | 5120 |
|  | 10+ copies | 22870 | 2210 |
| *Sorghum* | 1 copy | 9303 | 16995 |
| *bicolor* | 2-9 Copies | 9314 | 4594 |
|  | 10+ copies | 5000 | 580 |
| *Zea* | 1 copy | 5224 | 13691 |
| *mays* | 2-9 Copies | 18168 | 15072 |
|  | 10+ copies | 30950 | 2460 |

Supplementary Table 1

Number of syntenic and non-syntenic genes based on gene copy number within each species (copy number based on number of non-self BlastP hits within genome E<10^-10^ )

|  | Publication - genome | Genbank Genome assembly accession # | Genbank methylation data SRA |
| --- | --- | --- | --- |
| rice (Oryza sativa) | (Kawahara et al. 2013) | GCA_001433935.1 | SRX205347 |
| sorghum (Sorghum bicolor) | (Paterson et al. 2009) | GCA_000003195.3 | SRX1656933 |
| maize (Zea mays) | (Schnable et al. 2009) | GCA_000275765.1 | SRX120258 |
| purple false brome (Brachypodium distachyon) | (Vogel et al. 2010) | GCA_000005505.4 | SRX1656912 |
| mouse ear cress (Arabidopsis thaliana) | (Kaul et al. 2000) | GCA_000001735.2 | SRR342382 |
| poplar (Populus trichocarpa) | (Tuskan et al. 2006) | GCA_000002775.3 | SRX1656930 |
| peach (Prunus persica) | (Verde et al. 2013) | GCA_000346465.2 | SRX1656929 |
| grape (Vitis vinifera) | (Jaillon et al. 2007) | GCA_000003745.2 | SRX1656936 |
| tomato (Solanum lycopersicum) | (Sato et al. 2012). | GCA_900008105.1 | SRA046092 |

Supplementary Table 2

Source of raw data used in study.
